# Performance analysis and optimization of modulation techniques for underwater optical wireless communication in varied aquatic environments

**DOI:** 10.1038/s41598-025-18406-y

**Published:** 2025-09-17

**Authors:** M. Mokhtar Zayed, Mona Shokair

**Affiliations:** 1 Department of Communications, Faculty of Electronic Engineering, Menoufia University, Menoufia Governorate, Menouf City, Egypt; 2 Department of Communications and Computers Engineering, Higher Institute of Engineering, El-Shorouk Academy, El-Shorouk City, Egypt; 3 Department of Electrical Engineering, Faculty of Engineering, October 6 University, Giza Governorate, October 6 City, Egypt

**Keywords:** Underwater optical wireless communication (UOWC), Modulation techniques, Bit error rate (BER), Signal-to-Noise ratio (SNR), Spectral efficiency, Power efficiency, Turbulence effects., Electrical and electronic engineering, Lasers, LEDs and light sources, Optical physics

## Abstract

Underwater Optical Wireless Communication (UOWC) has emerged as a promising technology for enabling high-speed and low-latency data transmission in aquatic environments. However, the performance of UOWC systems is severely affected by absorption, scattering, turbulence, and background noise, making the choice of modulation techniques vital for ensuring reliable and efficient communication. This paper presents a comprehensive performance analysis of six prominent modulation schemes—On-Off Keying (OOK), Pulse Position Modulation (PPM), Quadrature Phase Shift Keying (QPSK), Differential Phase Shift Keying (DPSK), 32-Phase Shift Keying (32-PSK), and 64-Quadrature Amplitude Modulation (64-QAM)—under various underwater conditions. The study utilizes both LED and LD photo-sources (LED-PS and LD-PS) operating at a green wavelength of 520 nm, optimal for coastal and turbid harbor waters due to its lower attenuation characteristics. A silicon photomultiplier (SiPM-PD) with a receiver sensitivity of -53.4 dBm is employed for detecting low-power signals. Extensive numerical simulations evaluate key performance metrics including received power, Bit Error Rate (BER), Signal-to-Noise Ratio (SNR), channel capacity, and link range across different water types—pure, clear, coastal, and turbid—and turbulence intensities. This study offers the advantage of integrating multiple transmitter types, modulation schemes, and environmental factors, providing a comprehensive exploration of the trade-offs between communication range, transmitted power, and channel capacity. Results indicate that OOK enables the longest achievable communication distance (up to 123.73 m in pure seawater at BER = 10⁻⁵ with LD-PS), while high-order schemes such as 64-QAM achieve superior channel capacities (up to 53.23 bps/Hz), albeit at the cost of increased power and SNR requirements. The findings underscore a fundamental trade-off between range and Shannon spectral efficiency, guiding the selection of optimal modulation strategies for varied underwater applications, including environmental monitoring, underwater robotics, and defense communications.

## Introduction

 UOWC is rapidly emerging as a transformative technology for high-speed, low-latency data transmission in aquatic environments. As the demand for real-time communication and data exchange in underwater applications continues to grow, such as in environmental monitoring, underwater exploration, military operations, and remote sensing, the need for effective communication systems becomes even more crucial^1,2^. Unlike traditional methods such as acoustic or radio frequency (RF) communication, which suffer from inherent limitations such as low data rates, high latency, and poor reliability in challenging aquatic conditions, UOWC offers several key advantages^3–5^. These advantages include higher data rates, low power consumption, immunity to electromagnetic interference, and a significant reduction in the propagation delay, making it highly suitable for applications requiring large data throughput and real-time decision-making^3–5^. Despite its advantages, UOWC is significantly impacted by various underwater environmental factors, including absorption, scattering, turbulence, and background noise^6^. These factors reduce the effective communication range and impair the quality of the transmitted optical signals^7^. The optical signal attenuation caused by the properties of different water types, such as pure, clear, coastal, and turbid waters, presents a major challenge^8^. The presence of water turbulence further exacerbates signal degradation, leading to fluctuations in signal strength and increased error rates^9^. Given these environmental challenges, the selection of an appropriate modulation technique is paramount for ensuring reliable and efficient communication. Modulation techniques play a critical role in shaping the performance of UOWC systems by determining how data is encoded onto the optical carrier^10^. The choice of modulation scheme directly influences several important performance metrics, including communication range, channel capacity, SNR, BER, power efficiency, and spectral efficiency^11,12^. The task of selecting the optimal modulation scheme is complex due to the trade-offs involved between these metrics, particularly the balance between communication range and the achievable data rate^13^. For instance, while high-order modulation schemes such as 64-QAM can offer superior channel capacity, they typically require a higher SNR and more power, making them less suitable for long-range communication. On the other hand, simpler schemes like OOK can provide longer communication distances but with a lower data rate^10^. Given these challenges, a comprehensive performance analysis of different modulation techniques under varied underwater conditions is crucial to optimize UOWC systems for real-world applications. This paper aims to conduct an extensive evaluation of six prominent modulation schemes—OOK, PPM, QPSK, DPSK, 32-PSK, and 64-QAM—under a range of underwater conditions. This analysis will evaluate the modulation schemes in terms of key performance metrics such as communication range, capacity, power efficiency, and Shannon spectral efficiency, and how these metrics are influenced by the type of water (pure, clear, coastal, and turbid) and varying turbulence intensities^8,10^.

### Motivation

The motivation for this research stems from the increasing need to enhance communication capabilities in underwater environments, where traditional communication technologies such as acoustic and RF systems face significant challenges. These methods suffer from limitations like low data rates, high latency, and poor reliability, particularly in demanding conditions such as deep-sea exploration or turbulent waters^14,15^. As the demand for efficient underwater communication continues to grow, UOWC emerges as a promising alternative that can address many of these issues^16^. However, the performance of UOWC systems is highly sensitive to the choice of modulation scheme, making it crucial to evaluate and select the most effective options for diverse applications^17,28^. This study aims to identify the most Suitable modulation techniques for optimizing the performance of UOWC systems across a variety of underwater environments. With increasing reliance on underwater communication for applications Like environmental monitoring, underwater robotics, and defense communications, understanding how different modulation schemes perform under varying conditions is critical. For example, environmental monitoring may require long-range communication, where simpler modulation schemes Like OOK could be more appropriate. In contrast, high-capacity applications, such as real-time data streaming or underwater robotics, may benefit from more complex modulation techniques Like 64-QAM, which offer greater channel capacity but at the cost of higher power consumption^18^. By evaluating a range of modulation schemes, this study seeks to provide a detailed understanding of their trade-offs in terms of range, capacity, and efficiency, ultimately guiding the selection of the optimal modulation technique for each specific application.

### Key contributions

This paper presents the following key contributions:

1. **Comprehensive Modulation Comparison**: The study compares six widely used modulation schemes—OOK, PPM, QPSK, DPSK, 32-PSK, and 64-QAM—providing a thorough analysis of their performance across different underwater environments, including pure, clear, coastal, and turbid waters.
2. **Evaluation of Performance Metrics**: Key performance metrics, such as received power, BER, SNR, power efficiency, and Shannon spectral efficiency, are evaluated for each modulation scheme, offering insights into their suitability under various environmental conditions.
3. **Integration of Multiple Transmission Configurations**: The study integrates multiple transmitter types (LED and LD photo sources) and modulation schemes, offering a holistic view of the impact of different environmental factors on UOWC system performance.
4. **Analysis of Environmental Impact**: The effects of different water types and turbulence intensities on modulation performance are systematically analyzed, helping to identify the optimal modulation techniques for a wide range of aquatic environments.
5. **Optimization of Range and Efficiency**: A detailed investigation of the trade-offs between communication range and Shannon spectral efficiency is presented, providing guidance on how to select the best modulation technique based on application-specific requirements (e.g., long-range communication versus high-data-rate transmission).
6. **Guidance for Future UOWC Systems**: The findings serve as a valuable reference for researchers and engineers in the field of underwater communication, facilitating the development and deployment of high-performance UOWC systems for various applications, including marine exploration, disaster monitoring, and military operations.

The remainder of this paper is organized as follows: **Sect. 2** presents a comprehensive literature review, highlighting recent advancements and challenges in modulation techniques for underwater optical wireless communication. **Section 3** introduces the proposed system block diagram, detailing the key components and signal flow. **Section 4** provides the mathematical model and analytical framework used to evaluate the performance of various modulation schemes under different underwater conditions. In **Sect. 5**, extensive numerical simulations and discussions are presented, analyzing communication distance, power efficiency, BER, SNR requirements, and channel capacity across modulation formats. **Section 6** concludes the findings, emphasizing the trade-offs between distance and data rate. Finally, **Sect. 7** outlines potential future research directions, including the integration of adaptive modulation and AI-driven optimization techniques to enhance system performance in dynamic underwater environments.

## Literature reviews

Modulation techniques play a crucial role in the performance and reliability of UOWC systems, directly impacting factors such as BER, SNR, spectral efficiency, power consumption, and robustness to channel impairments. Due to the unique underwater environment, which is affected by absorption, scattering, and turbulence, selecting an optimal modulation scheme is essential for ensuring efficient data transmission. This section reviews existing research on UOWC modulation techniques, focusing on their comparative performance and optimization strategies for improving reliability in varying aquatic conditions.

The study in^19^ evaluated various modulation techniques for underwater optical wireless communication through modeling and simulation. Results showed that PPM is well-suited for low-power systems, while PSK offers superior bandwidth and error performance but with poor power efficiency. OOK, despite its simplicity, exhibited limitations in power efficiency and error control. DPSK demonstrated strong error resilience and high bandwidth but required significant power due to its optical interferometer-based receiver. FSK was found to be power-intensive and less desirable for underwater systems. The findings emphasized the need to balance water properties, wavelength selection, and modulation techniques to optimize underwater communication efficiency.

According to^20^, the study compared various intensity modulation techniques for underwater optical wireless communication, considering realistic system parameters. Results showed that PPM was the most energy-efficient, while DPIM offered better bandwidth efficiency and peak-to-average power ratio (PAPR) than PPM, though at the cost of higher demodulation complexity. OOK, despite its simplicity, was found to be less suitable for energy-constrained underwater environments. The findings highlighted the trade-offs between energy efficiency, bandwidth performance, and computational complexity in selecting optimal modulation schemes for underwater optical communication.

The authors of^21^ analyzed the performance of underwater optical wireless communication using various modulation techniques with an APD receiver under different Jerlov water types. Results showed that H-QAM provided superior performance, particularly in clear waters, due to its higher spectral efficiency. Si APD demonstrated better signal reception than Ge APD, especially in low-chlorophyll environments. Increased chlorophyll concentration reduced received optical power and SNR, affecting transmission distance. BER analysis confirmed that QAM outperformed other modulation schemes, with Si APD achieving a 13 dBm and 12 dBm advantage over Ge APD at 25 m and 50 m, respectively.

In^22^, the study analyzed the impact of water attenuation on underwater optical wireless communication using an LOS model. Results showed that OOK and 2DPSK provided better performance, particularly in Jerlov Type I water at 450 nm, which enabled longer transmission distances than 600 nm. Increased chlorophyll concentration reduced received power and SNR, limiting communication range. A larger receiver aperture (0.1 m²) improved reliability over extended distances. Findings confirmed that 450 nm is the optimal wavelength for UOWC due to lower attenuation and higher transmission efficiency.

Ref^23^. evaluated the performance of a UOWC system using a red laser and different modulation techniques. OFDM demonstrated superior performance over OOK and QPSK, achieving lower BER and higher bandwidth efficiency under the same turbulence conditions. Results showed that OFDM is preferable for low-energy transmissions while maintaining acceptable BER, making it a viable solution for high-speed, long-distance underwater communication.

As noted in^24^, the study analyzed the performance of PSK, DPSK, PAM, and QAM modulation techniques for underwater free-space optical (UW-FSO) communication across different water types. QAM demonstrated the best overall performance, achieving lower BER across all water conditions. Pure seawater allowed the longest communication distance due to minimal attenuation, while turbid harbor water resulted in the worst performance due to high optical wave attenuation.

The study in^25^ evaluated UOWC system performance considering attenuation, turbulence, pointing errors, and angle-of-arrival (AOA) fluctuations using analytical models. Results showed that heterodyne detection outperformed IM/DD, with M-PSK achieving better BER than M-QAM. Increasing receiver FoV and aperture size improved system performance, while asymmetric beam misalignment reduced outage probability. Strong absorption in Jerlov water II led to the worst performance, and narrower beamwidths enhanced ergodic capacity.

The authors of^26^ evaluated the BER performance of a single-channel UOWC system using different modulation techniques at 532 nm with an Si APD receiver. Results showed that 4-QAM provided the best BER performance, especially in pure seawater, where a BER of 10⁻⁹ was achieved at − 9.2 dBm for a 25 m link, requiring higher power in clear ocean and coastal waters.

In^27^, the study empirically compared modulation schemes in still and turbulent water, showing that SIM and PPM were more resilient to turbulence-induced fading, with SIM achieving higher data rates. While PPM was spectrally inefficient, pairwise coding (PWC) applied to SIM improved data rates in still water, achieving 5.2 Gbps, but performed worse in turbulence. Bit and power loading significantly improved SIM performance in turbulence by adapting modulation order based on channel state information.

As noted in^28^, the study designed and evaluated an underwater optical communication Link for up to 130 m at 40 m depth, achieving 15 Gbps using DQPSK and OFDM-QAM. Results showed that DQPSK outperformed other schemes in eye-opening, Q-factor, and Link reach, making it robust against phase shifts and fading. OFDM-QAM proved effective for distances up to 123 m, mitigating power penalties from polarization mode dispersion.

According to^29^, the study applied CNNs for modulation recognition in UOWC, focusing on 64-QAM and 32-PSK. The CNN model outperformed traditional methods, achieving 100% accuracy with a minimal loss rate of 1.82 × 10⁻⁶. Performance metrics, including precision, recall, F1-score, and AUC, all reached 100%, demonstrating the model’s effectiveness. These results highlight CNN’s potential for enhancing IoUT communication reliability, making OWC a superior alternative to acoustic and RF technologies for high-speed, data-intensive underwater applications.

The authors of^30^ investigated 4 × 4 MIMO-based UOWC for IoUT applications, demonstrating its superiority over SISO and SIMO (1 × 1 and 1 × 4) configurations. The MIMO system significantly enhanced data rates, communication range, and reliability by mitigating signal attenuation, scattering, and turbulence. Results showed a 70.9% reduction in transmitted power, an 18-fold increase in Link range, and a 2x improvement in channel capacity compared to SISO. Higher-order modulation schemes Like 64-QAM improved capacity, while OOK offered better energy efficiency. These findings highlight MIMO’s potential for high-speed, robust underwater communication, making it ideal for environmental monitoring, maritime security, and IoUT applications.

The study in^31^ focused on developing a high-capacity UOWC system using dual-polarization states and 16-QAM with OFDM modulation. A single 532 nm laser diode was employed to transmit orthogonally polarized signals along the X and Y axes, achieving a total data rate of 80 Gbps. System performance was assessed using BER, EVM, and constellation diagrams across ten water types. Results showed that low-attenuation waters Like pure water and Jerlov I enabled longer transmission distances up to 13.93 m, while high-attenuation waters Like Harbor I Limited the range to around 3 m under the same performance thresholds.

In 32, the study compared NRZ, AMI, and CSRZ modulation formats in a 10 Gbps UOWC system using a 532 nm laser across five water types. NRZ showed the best performance, offering the longest ranges and lowest BER, while CSRZ performed the worst. In pure seawater, NRZ achieved 28 m at BER ~ 10⁻⁹ and − 21 dBm, compared to 26.3 m for CSRZ. In Harbor II, ranges dropped to 4.06 m (NRZ) and 3.96 m (CSRZ). Performance varied with modulation type and water attenuation.

As noted in^33^, the study analyzed various UVLC modulation schemes, including NRZ-OOK, PPM, QAM-CAP, and OFDM, under underwater challenges Like scattering and turbulence. OFDM and space-domain index modulation showed superior performance, enabling data rates over 30 Gbps at distances up to 21 m. The findings highlighted the importance of choosing optimal modulation for improved BER, spectral efficiency, and system robustness in dynamic aquatic environments.

The authors of^34^ proposed a high-speed UOWC system using three OAM beams (LG₀,₀, LG₀,₂₀, LG₀,₅₀) from a single 532 nm laser, each carrying 10 Gbps for a total of 30 Gbps. Performance was analyzed across five Jerlov water types using BER, Q-factor, and eye diagrams. The longest range of 22 m was achieved in JI water due to low attenuation, while JIII allowed only 4.2 m. All transmissions met reliable reception criteria (Q > 4, log (BER) < − 5), confirming the potential of OAM multiplexing for high-capacity underwater links.

According to^35^, the study focused on implementing the fixed right shift (FRS) code in an OCDMA-based UOWC system using a 532 nm laser diode. The system was evaluated across five Jerlov water types with varying chlorophyll concentrations and at different data rates per channel (2.5, 5, and 10 Gbps). Results showed that increasing the data rate and water attenuation led to higher BER, reduced Q-factor, and narrower eye diagram openings. In Jerlov type I water, each 10 Gbps channel achieved a maximum range of 35 m with log (BER) ≤ − 6.33 and Q-factor > 4.9, while ranges dropped to 12 m in JII and 5.15 m in JIII. The system achieved a total capacity of 30 Gbps using three parallel channels.

Ref^36^. focused on developing a high-speed, long-range UOWC system using space division multiplexing (SDM) with distinct Hermite-Gaussian (HG) modes. Four 20 Gbps NRZ-encoded signals were transmitted over separate HG modes using a single-wavelength laser, achieving a total data rate of 80 Gbps over an 1800 m underwater link. The system’s performance was analyzed under varying wavelengths, aperture diameters, attenuation coefficients, and Link ranges. Results indicated that 850 nm offered the best performance, and optimizing the spatial aperture to 50 μm enabled stable BER and extended transmission up to 1950 m, even under high attenuation.

## The proposed system block diagram

**Fig. 1:**
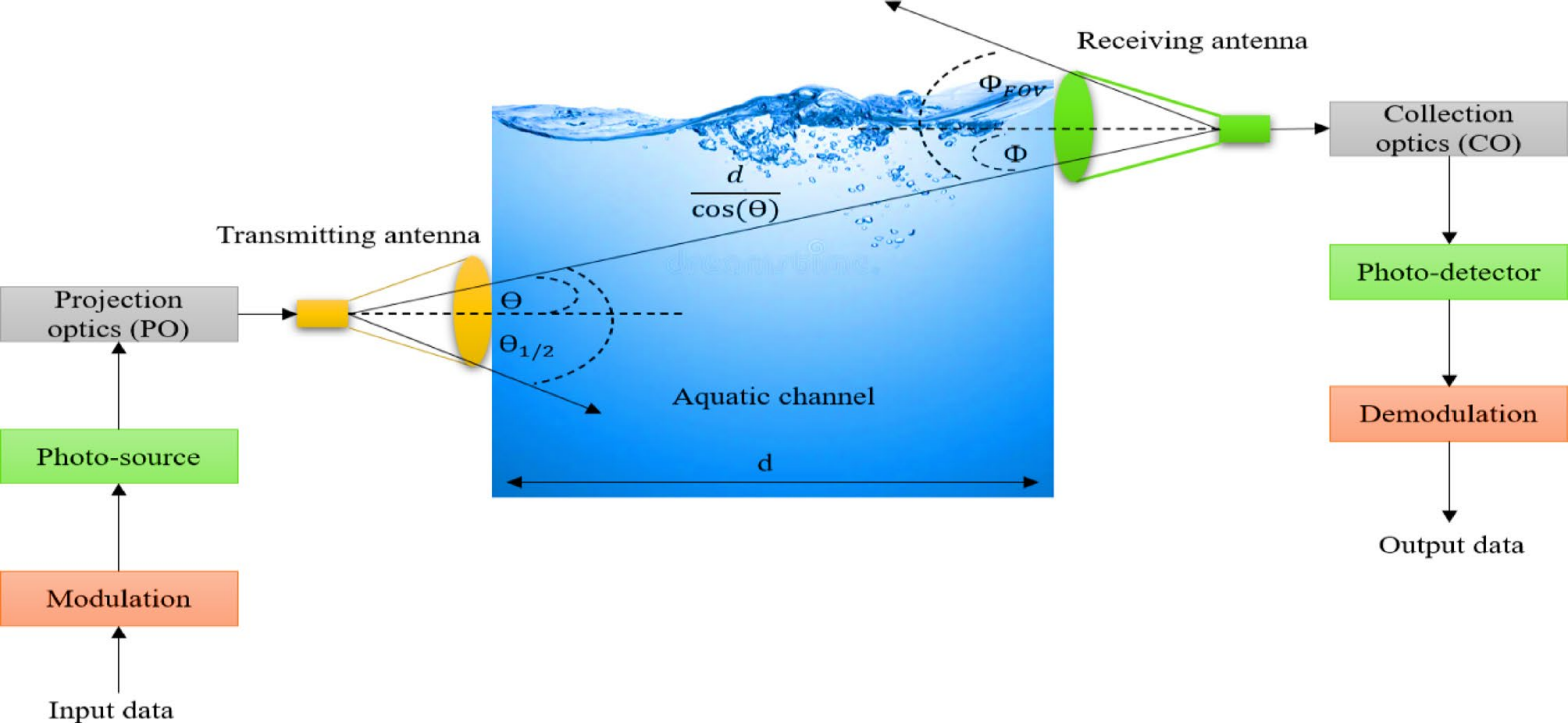
The proposed system block diagram.

A typical UOWC system integrates several critical modules to ensure robust data transmission and recovery in underwater environments. The process begins with raw input data, which undergoes modulation using schemes such as OOK, PPM, QPSK, DPSK, 32-PSK, and 64-QAM, depending on system design and application requirements. Once modulated, the optical signal is generated through photo sources—either LEDs or laser diodes. To enhance the directionality and focus of the light beam, optical projection systems are employed, typically utilizing beam expanders based on either Galilean or Keplerian designs^15,38^. The modulated Light then propagates through the aquatic medium, which imposes various degradations such as scattering, absorption, and optical turbulence. These environmental effects are often modeled using statistical distributions like log-normal, gamma-gamma, and Weibull to accurately capture the dynamic nature of the underwater optical channel. At the receiving end, optical collection systems gather the distorted light signal. Depending on the system configuration, these optics may include Hemispherical lenses with a wide 90° Field of View (FOV) or Compound Parabolic Concentrators (CPCs) with a narrower 30° FOV^4,5^. The collected signal is then detected by a SiPM, known for its exceptional sensitivity and ability to detect low-intensity signals, with a typical receiver sensitivity around − 53.4 dBm^37,38^. Finally, the received optical signal is passed through a demodulation unit that reconstructs the transmitted data, thus retrieving the original input information. Figure 1 illustrates the sequential arrangement of these components in a standard UOWC system.

This paper investigates the use of underwater visible light communication (VLC) for enabling uplink data transmission in aquatic environments. The communication system is anchored by a transmitting source node positioned on the ocean floor. This node emits an optical beam directed upward toward the water surface. The characteristics of the optical beam are defined by two parameters: the beam divergence angle (*θ*) and the semi-angle at half power (*θ₁/₂*), which influence how the light spreads through the medium. Positioned at the water’s surface, the sink node serves as the receiving unit. It is aligned to intercept the incoming light beam at a specific incident angle (*ϕ*). The receiver features a limited field of view (*ϕ*_*FOV*_), which governs its angular sensitivity and plays a crucial role in determining the range over which reliable communication can be maintained. The effective communication distance (*d*) between the source and sink nodes depends on both the relative spatial alignment and these angular parameters, as they interact within the constraints of the underwater optical propagation environment.

## The proposed mathematical model analysis

The mathematical modeling of UOWC modulation techniques is critical for assessing system performance under varying channel conditions. The key parameters influencing signal transmission include absorption, scattering, turbulence, noise, and attenuation, which significantly impact the received power, BER, SNR, power efficiency, and Shannon spectral efficiency.

### Channel attenuation model in UOWC

**Fig. 2:**
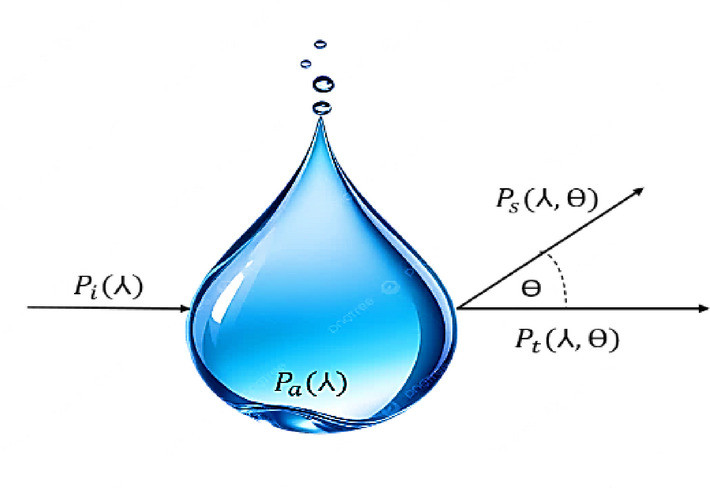
The geometry of optical beam transmission in underwater environments^39^.

The attenuation of optical signals in underwater environments is governed by the Beer-Lambert law, which defines the received power ​$\:{P}_{r}$ as:

1 $$\:{P}_{r}={P}_{t}{e}^{-c\left(\lambda\:\right)d}$$

where $\:{P}_{t}$ is the transmitted optical power (W), $\:c\left(\lambda \right)$ is the total attenuation coefficient (m^−1^), *d* is the transmission distance (m), and $\:{P}_{r}\:$is the received optical power (W). The attenuation coefficient $\:c\left(\lambda\right)$ consists of absorption and scattering contributions^38,41^:

2 $$\:c\left(\lambda\right)=a\left(\lambda\:\right)+b\left(\lambda\:\right)$$

where $\:a\left(\lambda\:\right)\:$is the absorption coefficient (m^−1^), $\:b\left(\lambda\:\right)\:$is the scattering coefficient (m^−1^), and $\:\lambda\:\:$is the operating optical wavelength (e.g., 450 nm, 520 nm). For different water types: Clear Water: α ≈ 0.1–0.30 m^−1^, Coastal Water: α ≈ 0.4–0.70 m^−1^, and Turbid Water: α > 1.0 m^−1^. The two primary physical mechanisms responsible for signal attenuation in underwater channels are absorption and scattering, as illustrated in Fig. 2.

Table 1 presents the standard values of *a(λ)*, *b(λ)*, and *c(λ)* for different water types, with an emphasis on the green spectrum at a wavelength of λ = 520 nm, applicable to both LED and LD photo-sources.

**Table 1:** Typical values of *a(λ)*, *b(λ)*, and *c(λ)* for various types of water at a wavelength of ⅄ = 520 nm^15,29,30^.

Types of water	Absorption coefficient $\:\mathbf{a}\left(\varvec{\uplambda}\right)\:\left({\mathbf{m}}^{-1}\right)$	Scattering coefficient $\:\mathbf{b}\left(\varvec{\uplambda}\right)\:\left({\mathbf{m}}^{-1}\right)$	Extinction coefficient $\:\mathbf{c}\left(\varvec{\uplambda}\right)\:\left({\mathbf{m}}^{-1}\right)$
Pure Sea Water	0.04418	0.0009092	0.0450892
Clear Ocean Water	0.08642	0.01226	0.09868
Coastal Ocean Water	0.2179	0.09966	0.31756
Turbid Harbor Water	1.112	0.5266	1.6386

**Fig. 3:**
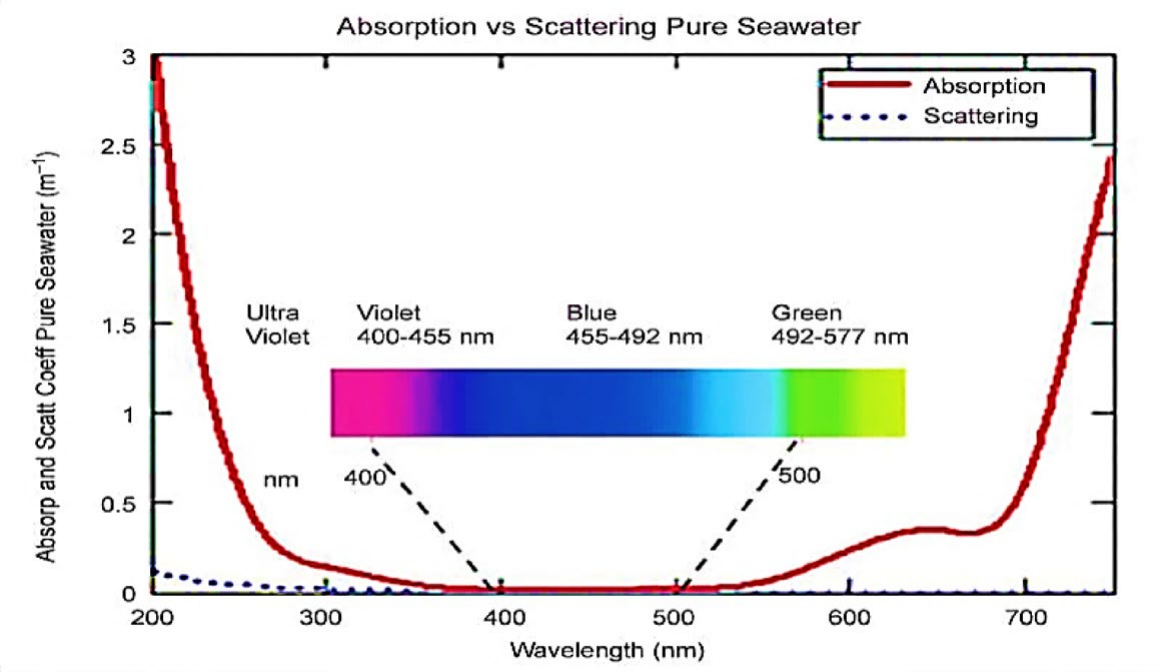
The relationship between the absorption and scattering coefficients of light and the wavelength in pure seawater^30,40^.

The green wavelength (520 nm) is ideal for underwater optical communication in coastal and turbid waters due to its lower attenuation. Compared to other wavelengths, green light experiences less absorption and scattering, allowing it to travel farther and maintain signal strength, even in turbid conditions with suspended particles. This makes it more efficient for longer-range transmission in such environments^4,5,38^. Figure 3 shows how the absorption and scattering coefficients of light in pure seawater change with wavelength, as reported in^30,40^.

### Noise model in UOWC

The total noise is expressed as:

3 $$\:{N}_{total}={N}_{shot}+{N}_{thermal}+{N}_{dark}+{N}_{sig}+{N}_{rin}$$

where $\:{N}_{total}$ is the total noise power, including: Background shot noise $\:{N}_{shot}$​, Thermal noise $\:{N}_{thermal}$​, Dark current noise $\:{N}_{dark}$, Signal shot noise $\:{N}_{sig}$​, Relative intensity noise (RIN) $\:{N}_{rin}$​ (for Laser Diodes).

- Signal Shot Noise

4 $$\:{N}_{sig}=2qR{P}_{r}B$$

where *q* is the electron charge (1.6 × 10^−19^ C), *R* is the receiver responsivity (A/W), and B is the Receiver bandwidth (Hz).

(b) Background Shot Noise

5 $$\:{N}_{shot}=2qR{P}_{bg}B$$

where $\:{P}_{bg}\:$is the background optical power (W).

(c) Thermal Noise

6 $$\:{N}_{thermal}=\frac{4KTB}{{R}_{L}}\:\:$$

where *K* is the Boltzmann’s constant (1.38 × 10^−23^ J/K), *T* is the temperature (K), and ​$\:{R}_{L}$ is the receiver load resistance (Ω).

(d) Dark Current Noise

7 $$\:{N}_{dark}=2q{I}_{d}B$$

where $\:{I}_{d}$ is the dark current (A).

(e) Relative Intensity Noise (RIN)

8 $$\:{N}_{rin}=\text{R}\text{I}\text{N}\cdot\:{P}_{r}^{2}\:\text{}\text{B}$$

where *RIN* is the relative intensity noise coefficient (Hz^−1^).

### Turbulence model in UOWC

The Log-Normal turbulence model in UOWC describes the fluctuations in signal intensity due to turbulence. The turbulence-induced fading factor, *γ*, is the ratio of instantaneous to mean received intensity and is expressed as:

9 $$\:\gamma\:=\frac{I}{\langle I \rangle}\:$$

where *I* is the instantaneous intensity and ⟨I⟩ is the average intensity. The probability density function (PDF) of *γ* follows a log-normal distribution^42^:

10 $$\:P\left(\gamma\:\right)=\:\frac{1}{\gamma\:\sqrt{2\pi\:{\sigma\:}^{2\:}}}\:{exp}\left(-\frac{{\left(\text{l}\text{n}\left(\gamma\:\right)-\mu\:\right)}^{2}}{2{\sigma\:}^{2}}\right)\:$$

where *µ* and σ^2^ are the mean and variance of ln(γ), respectively. This model captures the impact of turbulence on optical signal quality in UOWC systems.

### The received power in UOWC

**Fig. 4:**
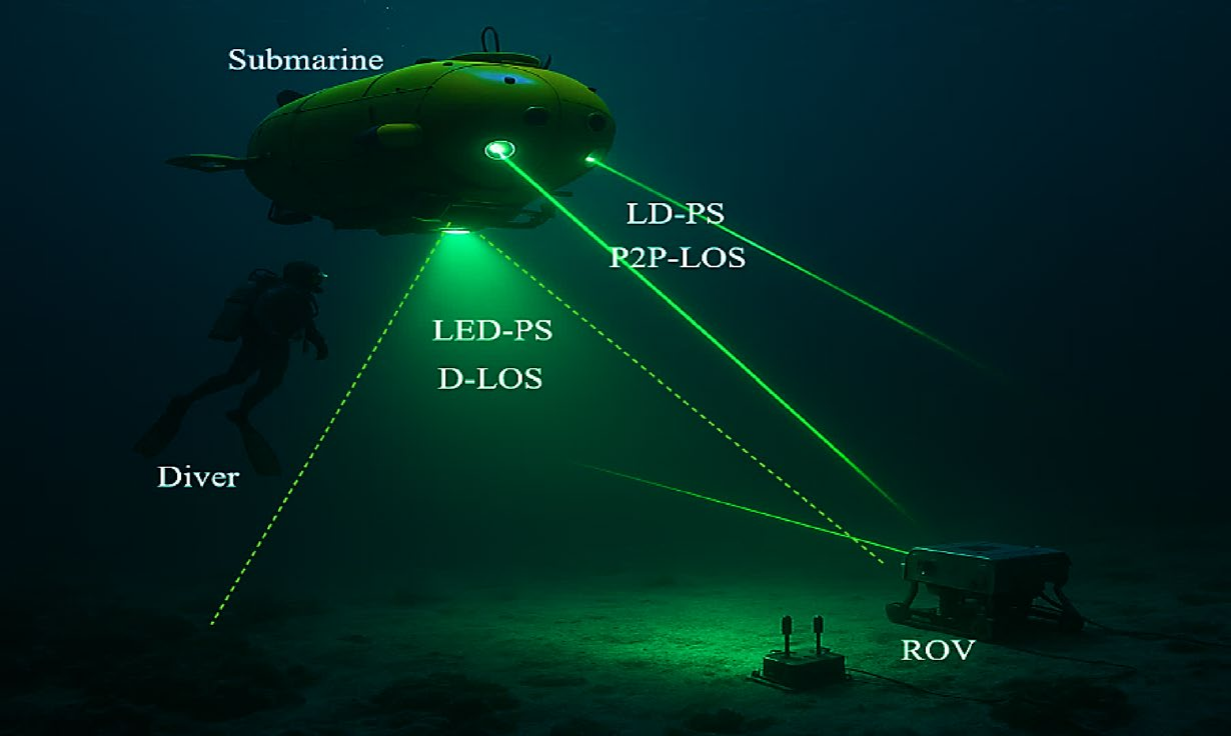
P2P-LOS and D-LOS link configurations in UOWCs.

In a point-to-point line-of-sight (LOS) configuration using a LD-PS, the received optical power is significantly higher due to the highly focused beam, which reduces scattering and enhances power efficiency. This enables long-range communication with minimal energy loss. However, LD-PS systems require precise alignment between the transmitter and receiver, making them vulnerable to misalignment and beam wander caused by underwater turbulence. In contrast, a diffuse LOS setup with an LED-PS results in lower received optical power due to the wider beam divergence, which increases scattering and attenuation. Although this reduces power efficiency and restricts the communication range, it offers better coverage and more relaxed alignment requirements, making it more resilient in dynamic underwater environments^4,5^. Figure 4 illustrates the configurations of P2P-LOS and D-LOS links in UOWC systems.

#### Received power for LED-PS

In an optical wireless communication system utilizing LEDs, the transmitted light typically features a wider beam divergence. The optical power received at the photodetector can be expressed as^42^:

11 $$\:{P}_{r,\:\:\:LED-PS}={\:P}_{t\:}{\eta\:}_{t\:}{\eta\:}_{r}\:{e}^{-c\left(\lambda\:\right)d}\:{(\frac{\left(m+1\right)}{2\pi\:}{cos}}^{m}(\theta\:\left)\right){\:\:G}_{TXPO}\:{G}_{RXCO}\left(\phi\:\right)\:{T}_{S}\left(\phi\:\right)\:\:{F}_{turb\:\:\:}\frac{{A}_{PD}{cos}(\phi\:)}{2\pi\:{d}^{2}(1-cos\theta)}\:\varPi\:\left(\frac{\phi\:}{{\phi\:}_{FOV}}\right)$$

where

12 $$\:\varPi\:\left(\frac{\phi\:}{{\phi\:}_{FOV}}\right)=\left\{\begin{array}{c}\text{1}\text{}\text{}\text{}\text{}\text{,}\text{}\text{}\text{}\text{}\text{}\text{}\text{}\text{}\text{}\text{}\text{}\frac{\phi\:}{{\phi\:}_{FOV}}\le\:1\\\:\text{}\text{}\text{}\text{}\text{0}\text{}\text{}\text{}\text{}\text{,}\text{}\text{}\text{}\text{}\text{}\text{}\text{}\text{}\text{}\text{}\text{}\text{}\text{}\text{o}\text{t}\text{h}\text{e}\text{r}\text{w}\text{i}\text{s}\text{e}\text{}\text{}\text{}\end{array}\right.\:$$

Here, $\:{\:P}_{t\:}$denotes the transmitted optical power, $\:{\eta\:}_{t\:}$and $\:{\eta\:}_{r}$​ represent the efficiencies of the transmitter and receiver, respectively. The total attenuation coefficient *c(λ)*, which encompasses both absorption and scattering effects, quantifies the channel loss. The gain of the transmitter’s projection optics is indicated by *G*_*TXPO*_, and the receiver’s collection optics gain, dependent on the angle *ϕ*, is given by *G*_*RXCO*_*(ϕ).* The term *T*_*S*_*(ϕ)* reflects the transmission gain of the optical bandpass filter. Fading due to underwater turbulence is captured by *F*_*turb*_. The photodetector’s effective aperture area is denoted by A_PD_​, and *d* corresponds to the transmission distance. The LED’s emission profile is described by the Lambertian order mm, which is calculated as:

13 $$\:m=\:\frac{-\text{ln}\left(2\right)}{\text{l}\text{n}\left(cos{\theta}_{\frac{1}{2}}\right)}\:$$

The parameter $\:{\theta}_{\frac{1}{2}}$ defines the half-power semi-angle of the optical source. The gain associated with the receiver’s collection optics can be formulated as:

14 $$\:{G}_{RXCO}\left(\varPhi\:\right)=\left\{\begin{array}{c}\frac{{n}^{2}}{{{sin}}^{2}{\varPhi\:}_{FOV}}\text{,}\text{}\text{}\text{}\text{}\text{}\text{}\text{}\text{}\text{}\text{}\text{}\text{}\text{}\text{}\text{}\text{}\text{}\text{}\text{}\text{}\text{}\text{}\text{0}\le\:\varPhi\:\le\:{\varPhi\:}_{FOV}\text{}\text{}\text{}\text{}\text{}\text{}\text{}\text{}\text{}\text{}\text{}\text{}\\\:\text{0}\text{}\text{}\text{}\text{}\text{}\text{}\text{}\text{}\text{}\text{}\text{,}\text{}\text{}\text{}\text{}\text{}\text{}\text{}\text{}\text{}\text{}\text{}\text{}\text{}\text{}\text{}\text{}\text{}\text{}\text{}\text{}\text{}\text{}\text{}\text{}\text{}\text{}\text{}\text{}\text{}\text{}\text{}\text{}\text{}\text{}\text{}\text{}\text{}\text{}\text{}\text{}\text{}\text{}\text{}\text{}\text{}\text{}\text{}\text{}\text{}\text{}\text{}\varPhi\:>{\varPhi\:}_{FOV}\:\:\:\:\:\:\:\end{array}\right.$$

where *n* denotes the refractive index of the lens inside the optical concentrator. This study utilizes a compound parabolic concentrator (CPC) configured with a field of view *Φ*_*FOV*_*​=π/6*. For the case of a Galilean beam expander, the gain of the transmitter optics is defined as^15,38^:

15 $$\:{G}_{TXPO}=\frac{f2}{\left|f1\right|}=\frac{{D}_{o}}{{D}_{i}}\:$$

Here, *f*_*1*_ and *f*_*2*_ correspond to the focal lengths of the concave and convex lenses, respectively, while *D*_*i*_ and *D*_*o*_ denote the diameters of the input and output beams. In a similar manner, the optical gain for a Keplerian beam expander is given by^15,38^:

16 $$\:{G}_{TXPO}=\frac{f2}{f1}=\frac{{D}_{o}}{{D}_{i}}\:\:$$

where *f*_*1*_ represent the focal length of the convex lens at the input. To simplify the analysis, the optical narrow bandpass filter (BPF) transmission gain is assumed to remain approximately constant and is defined as *T*_*S*_*(φ)* = 1.

#### Received power for LD-PS

For a laser diode (LD)-based source, the beam divergence is much smaller compared to that of an LED. The received power can be expressed as^42^:

17 $$\:{P}_{r,\:\:\:LD-PS}={P}_{t\:}{\eta\:}_{t}{\:\eta\:}_{r}\:{e}^{-c\left(\lambda\:\right)d}\:{\:\:G}_{TXPO}\:{G}_{RXCO}\left(\phi\:\right)\:{T}_{S}\left(\phi\:\right){\:\:F}_{turb\:\:\:}\frac{{A}_{PD}{cos}(\phi\:)}{\pi\:{d}^{2}({tan}\theta\:{)}^{2}}\:\varPi\:\left(\frac{\phi\:}{{\phi\:}_{FOV}}\right)$$

All parameters maintain their previously defined meanings, with the key difference being the focused nature of the laser beam, which is represented by the denominator term$\:\:({tan}\theta\:{)}^{2}$.

### Signal-to-noise ratio (SNR) in UOWC

The SNR at the receiver is given by:

18 $$\:SNR=\frac{{P}_{r}}{{N}_{total}}$$

### Bit error rate (BER) analysis for modulation techniques

The BER for different modulation techniques is calculated based on the SNR.

- On-Off Keying (OOK)

OOK is a simple intensity modulation scheme. The BER for OOK under an Additive White Gaussian Noise (AWGN) channel is given by:

19 $$\:{BER}_{OOK}=Q\left(\sqrt{\frac{\gamma\:}{2}}\right)$$

Where γ is the signal to noise ratio (SNR) and the Q-function is calculated through this formula:

20 $$\:Q\left(x\right)=\frac{1}{\sqrt{2\pi\:}}\underset{x}{\overset{\infty\:}{\int\:}}{e}^{{-t}^{2}/2}\:dt$$

(b) Pulse Position Modulation (PPM)

PPM transmits data by shifting pulse positions within a symbol period. Its BER in case of 2-PPM is:

21 $$\:{BER}_{2-PPM}=Q\left(\sqrt{\gamma\:}\right)$$

(c) Phase Shift Keying (DPSK and QPSK)

In Differential Phase Shift Keying (DPSK), the BER is:

22 $$\:{BER}_{DPSK}=\frac{1}{2}\:{e}^{-\gamma\:}$$

For Quadrature Phase Shift Keying (QPSK), BER is:

23 $$\:{BER}_{QPSK}=Q\left(\sqrt{\gamma\:}\right)$$

QPSK provides double spectral efficiency compared to BPSK while maintaining similar energy efficiency.

(d) Phase Shift Keying (32-PSK)

The BER for M-PSK is:

24 $$\:{BER}_{M-PSK}\approx\:\frac{1}{{log}_{2}M}\:Q\left(\sqrt{2\gamma\:{log}_{2}M.{sin}^{2}\left(\frac{\pi\:}{M}\right)}\:\right)$$

For **32-PSK** (M = 32):

25 $$\:{BER}_{M-PSK}\approx\:\frac{1}{5}\:Q\left(\sqrt{10\gamma\:.{sin}^{2}\left(\frac{\pi\:}{32}\right)}\:\right)$$

(e) Quadrature Amplitude Modulation (64-QAM)

Higher-order QAM improves spectral efficiency but is more sensitive to turbulence. The BER for M-QAM is:

26 $$\:{BER}_{M-QAM}\approx\:\frac{4}{{log}_{2}M}\left(1-\frac{1}{\sqrt{M}}\right)\:Q\left(\sqrt{\frac{3\gamma\:{log}_{2}M}{M-1}\:}\:\right)\:$$

where M = 16 or 64. 16-QAM offers better SNR performance in clear waters, while 64-QAM is more susceptible to noise and scattering.

For **64-QAM** (M = 64):

27 $$\:{BER}_{64-QAM}\approx\:\frac{4}{6}\left(1-\frac{1}{\sqrt{64}}\right)\:Q\left(\sqrt{\frac{21\gamma\:}{63}\:}\:\right)\:$$

### Power efficiency vs. shannon spectral efficiency trade-Off

The power and Shannon spectral efficiency (upper bound) of modulation schemes are analyzed as:

Power efficiency measures the amount of information transmitted per unit of transmitted power, indicating how effectively the system uses power to transmit data. It is defined as:​

28 $$\:{\eta\:}_{p}=\frac{C}{{P}_{t}}\:\:$$

where $\:{\eta\:}_{p}$​ is the power efficiency in (bits/J), *C* is the channel capacity in (bps), and $\:{P}_{t}$​ is the transmitted optical power in (W). In UOWC systems, power efficiency is influenced by factors such as modulation scheme, optical source characteristics (e.g., LED or LD), and the receiver’s sensitivity. For instance, using a SiPM detector can enhance sensitivity, thereby improving power efficiency^43^.​.

Shannon Spectral efficiency (upper bound) quantifies the data rate transmitted per unit bandwidth, reflecting how efficiently the system utilizes the available frequency spectrum. It is given by:​

29 $$\:{\eta\:}_{s}=\frac{C}{B}={log}_{2}(1+SNR)$$

where $\:{\eta\:}_{s}$ is the Shannon spectral efficiency (upper bound) in (bps/Hz) and *B* is the bandwidth in (Hz). Modulation schemes Like 64-QAM offer higher spectral efficiency by transmitting more bits per symbol, though they may require higher SNR to maintain performance.

## Numerical Results Analysis and Discussions

Table 2 provides a comprehensive overview of the key simulation parameters used in this study.

**Table 2:** Simulation Parameters.

Parameter	Symbol	Value/Range	
Photo sources	-	LD-PS and LED-PS	
Operating Wavelength	*λ*	520 nm (green color)	
Transmitter Power	$\:{P}_{t}$	1 W	
BER	-	$\:{10}^{-5}$	
Transmission distance	*d*	1–100 m	
Bandwidth	*B*	1 MHz	
Transmitter efficiency	$\:{\eta\:}_{TX}$	0.9	
Transmitter semi-angle at half power for D- LOS	$\:{\Theta}_{1/2}$	60 °	
Lambertian order for D-LOS	*m*	1	
Transmitter light beam divergence angle	$\:{\theta}_{1/2}$	LED-PS	30°
		LD-PS	9°
Water type	-	Pure, clear, coastal, and turbid harbor waters	
Total attenuation coefficient [15,29,30]	*C (λ)*	0.0450892, 0.09868, 0.31756, and 1.6386 m^−1^	
Noise sources	-	Shot, thermal, dark, and RIN noise	
Noise model		AWGN	
Turbulence model	-	Log-normal fading distribution	
Receiver efficiency	$\:{\eta\:}_{RX}$	0.9	
Receiver light beam incident angle	*Ф*	LED-PS	15°
		LD-PS	5°
Receiver FOV angle	$\:{\varPhi\:}_{FOV}$	30° (CPC)	
Receiver Aperture Area	$\:{A}_{PD}$	1 mm^2^	
Refractive index of lens at PD	*n*	1.5	
Photo detector (PD)	-	SiPM-PD	
Receiver sensitivity [37,38]	$\:{P}_{S}$	−53.4 dBm	
Photodetector Responsivity	*R*	0.4–0.6 A/W	
Load resistance	$\:{R}_{L}$	1000 Ω	
Modulation scheme	-	OOK, PPM, DPSK, QPSK, 32-PSK, 64-QAM	
Simulation tool	-	Python	

**Fig. 5:**
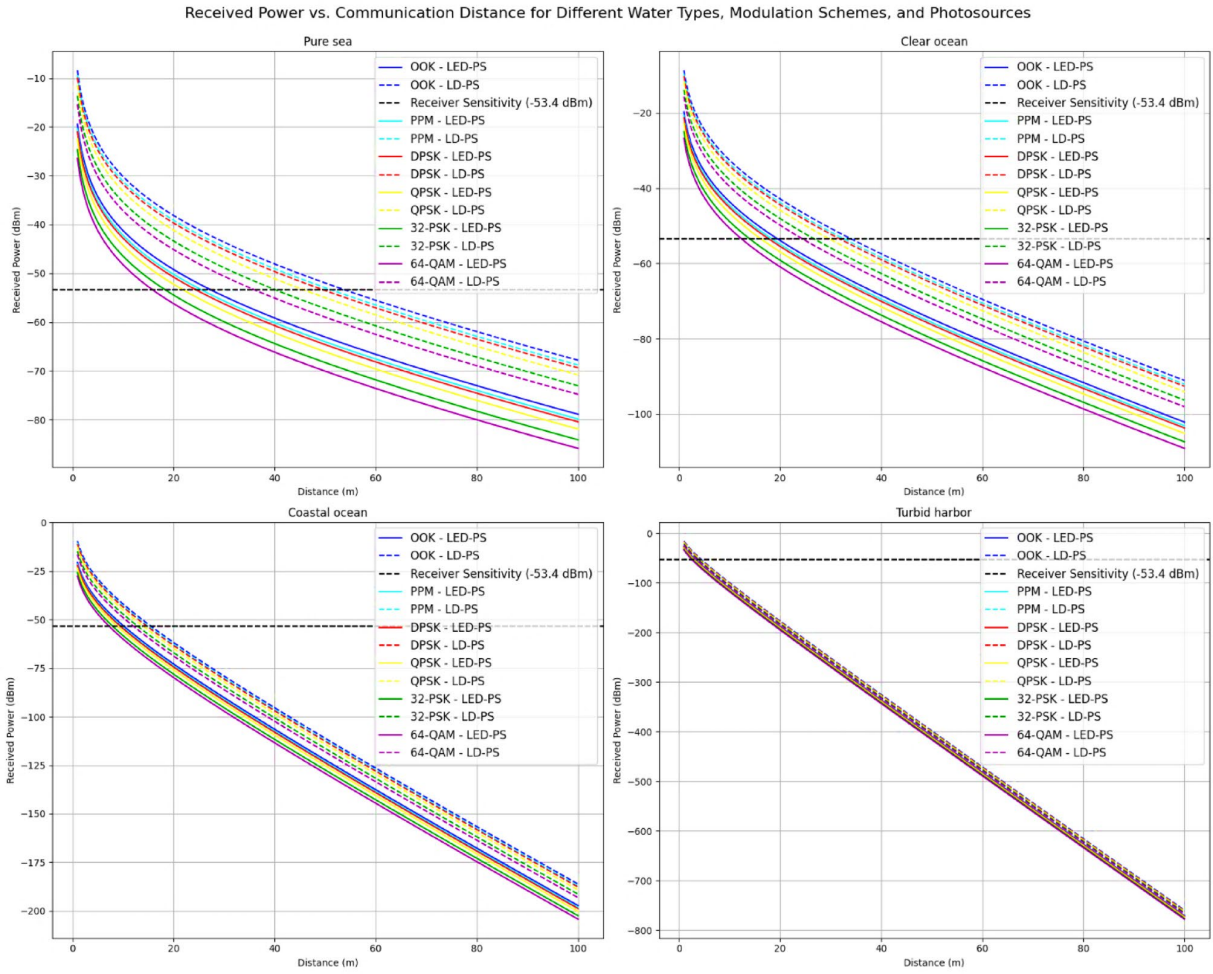
The received power vs. communication distance for various water types, modulation techniques, and transmitter types.

Figure 5 shows the variation of received optical power (dBm) with communication distance (meters) in different underwater environments: pure sea, clear ocean, coastal ocean, and turbid harbor. Modulation schemes such as OOK, PPM, DPSK, QPSK, 32-PSK, and 64-QAM are analyzed using both LED-PS and LD-PS photo sources. Received power decreases exponentially with distance, with LD-PS generally performing better than LED-PS. A receiver sensitivity threshold is indicated to mark the minimum power required for successful reception. The results highlight how water clarity affects optical signal attenuation.

The communication distance for various modulation techniques in different water types is analyzed in Table 3, considering a receiver sensitivity threshold of −53.4 dBm. The results reveal significant variations in achievable distances based on modulation schemes, photo-source types (LED-PS and LD-PS), and water conditions.

Across all water types, OOK consistently provides the highest communication distances, followed by PPM and DPSK. More complex modulation schemes, such as QPSK, 32-PSK, and 64-QAM, exhibit shorter communication ranges due to their higher spectral efficiency and increased susceptibility to attenuation and noise.

- In **pure sea water**, which has the least attenuation, OOK with LD-PS achieves the longest communication range of 53.87 m, whereas OOK with LED-PS reaches 27.37 m. The trend remains consistent across modulation schemes, with LD-PS outperforming LED-PS in all cases due to its higher power efficiency and narrower beam divergence, which minimizes scattering losses. The highest-order modulation scheme, 64-QAM, achieves the shortest distance of 36.03 m for LD-PS and 15.86 m for LED-PS, demonstrating the trade-off between data rate and transmission distance.
- In the **clear ocean**, which has slightly higher attenuation compared to pure sea water, the communication distances decrease for all modulation techniques. OOK with LD-PS reaches a maximum distance of 33.97 m, while 64-QAM achieves only 24.38 m. The trend of decreasing distance with increasing modulation complexity is evident, with LD-PS consistently outperforming LED-PS due to its superior focusing capability.
- In the **coastal ocean**, which exhibits even higher levels of scattering and absorption, the communication distances drop further. OOK with LD-PS achieves 15.50 m, while LED-PS reaches only 10.14 m. The higher-order modulation schemes, such as 64-QAM, experience significant attenuation, with a communication distance of only 12.03 m for LD-PS and 7.22 m for LED-PS. The reduction in transmission distance highlights the impact of water clarity on optical signal propagation.
- The most challenging environment is the **turbid harbor**, where high levels of suspended particles and impurities drastically Limit optical transmission. Here, OOK with LD-PS achieves a maximum range of just 4.51 m, while 64-QAM reaches a mere 3.75 m. The LED-PS performance is even more restricted, with OOK achieving only 3.33 m and 64-QAM just 2.63 m. These results underscore the severe impact of high turbidity on underwater optical wireless communication, where even the most power-efficient modulation schemes struggle to maintain long-distance transmission.

Overall, the results emphasize the importance of selecting an appropriate modulation scheme and photo-source type based on the underwater environment. LD-PS consistently provides greater communication distances than LED-PS due to its reduced beam divergence and higher optical power concentration. Simultaneously, simpler modulation schemes such as OOK and PPM offer better reach, while higher-order schemes, despite their increased data rates, suffer from significant transmission limitations. These findings are critical for designing underwater optical communication systems optimized for specific environmental conditions.

**Table 3:** Communication distances for various modulation techniques, water types, and photo-sources at receiver sensitivity − 53.4 dBm.

Water Type	Modulation Type	LED-PS Distance (m) at $\:{\varvec{P}}_{\varvec{S}}$ = −53.4 dBm	LD-PS Distance (m) at $\:{\varvec{P}}_{\varvec{S}}$ = −53.4 dBm
Pure Sea Water	OOK	27.37	53.87
	PPM	25.52	51.19
	DPSK	24.45	49.62
	QPSK	21.90	45.75
	32-PSK	18.37	40.18
	64-QAM	15.86	36.03
Clear Ocean	OOK	19.44	33.97
	PPM	18.35	32.56
	DPSK	17.71	31.73
	QPSK	16.16	29.68
	32-PSK	13.96	26.67
	64-QAM	12.34	24.38
Coastal Ocean	OOK	10.14	15.50
	PPM	9.71	15.00
	DPSK	9.46	14.70
	QPSK	8.83	13.97
	32-PSK	7.91	12.87
	64-QAM	7.22	12.03
Turbid Harbor	OOK	3.33	4.51
	PPM	3.23	4.40
	DPSK	3.17	4.34
	QPSK	3.02	4.18
	32-PSK	2.80	3.94
	64-QAM	2.63	3.75

**Fig. 6:**
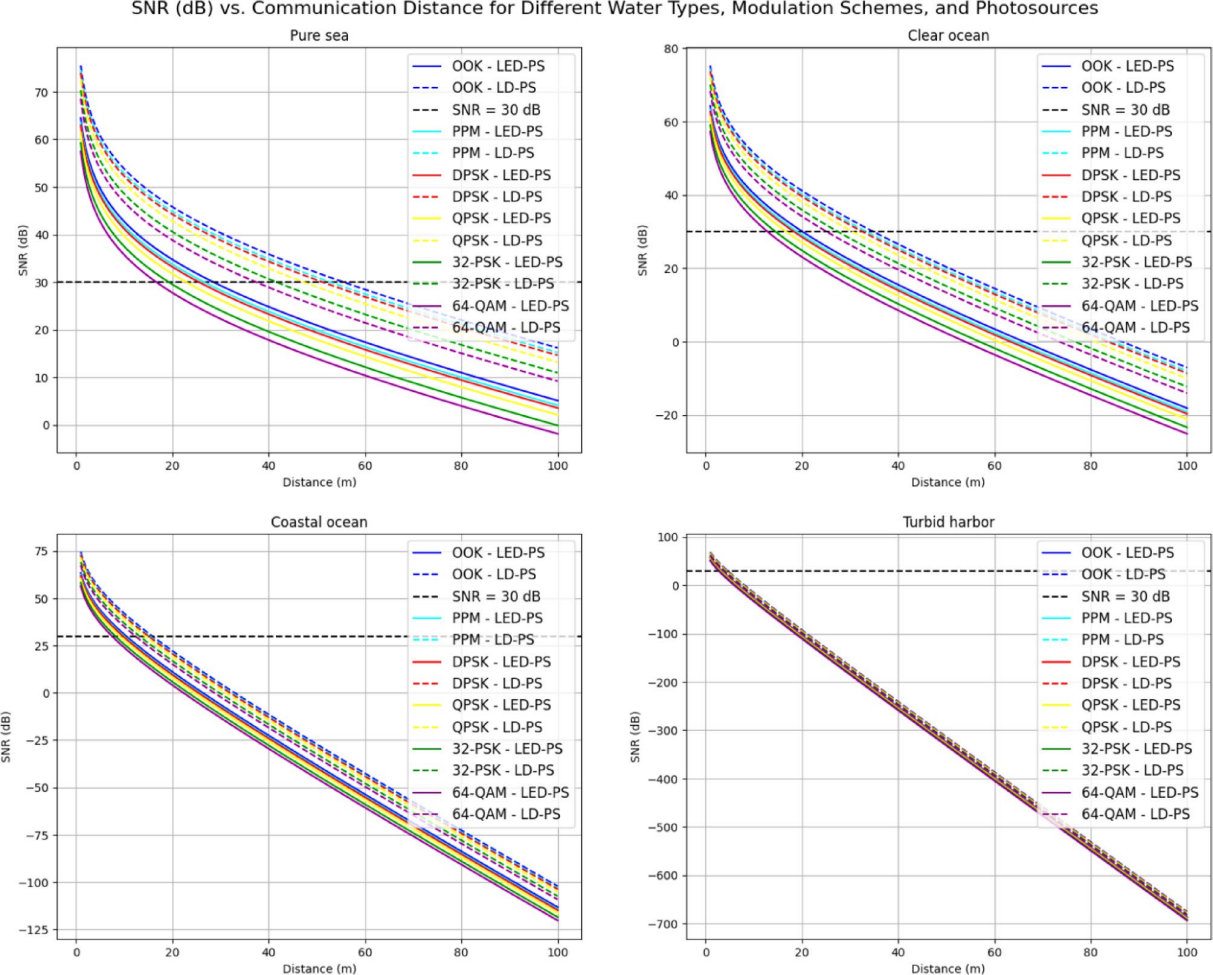
SNR vs. communication distance for various water types, modulation techniques, and transmitter types.

Figure 6 presents the relationship between SNR (dB) and communication distance for various modulation schemes in the same underwater environments. It compares the performance of OOK, PPM, DPSK, QPSK, 32-PSK, and 64-QAM using both LED-PS and LD-PS. A dashed black Line denotes the SNR threshold of 30 dB. The results show a general decline in SNR with increasing distance, and each modulation scheme displays different performance levels, reflecting trade-offs between signal quality and transmission range.

Table 4 presents the communication distances at an SNR of 30 dB for various modulation techniques, water types, and photo-sources. The data highlights how the choice of modulation type and the water environment significantly influence the achievable communication distance for both LED-PS and LD-PS systems.

- In **pure seawater**, the longest communication distance is observed with the OOK modulation for both LED-PS and LD-PS, with distances of 28.51 m and 55.50 m, respectively. As the modulation scheme becomes more complex, such as with 64-QAM, the distance decreases substantially. For LED-PS, the distance drops to 16.66 m, and for LD-PS, it decreases to 37.36 m. This indicates that simpler modulation schemes, Like OOK, provide better range, Likely due to their lower complexity and energy requirements, while more advanced schemes Like 64-QAM, though offering higher data rates, incur a greater distance penalty.
- The trend continues in the “**Clear Ocean**” water type, where the distances for OOK and 64-QAM are 20.10 m and 12.86 m for LED-PS, and 34.81 m and 25.12 m for LD-PS. As expected, more complex modulation types result in shorter communication ranges, demonstrating a trade-off between data rate and range. This is further emphasized as we move to “Coastal Ocean” and “Turbid Harbor” environments, where both the LED-PS and LD-PS distances continue to decrease across all modulation types.
- In the “**Coastal Ocean**” environment, the LED-PS distances range from 10.40 m for OOK to 7.44 m for 64-QAM, while LD-PS distances range from 15.80 m for OOK to 12.30 m for 64-QAM. This reduction in communication distance can be attributed to the increasing attenuation and scattering effects in such waters, which degrade the signal over longer distances.
- Finally, in “**Turbid Harbor**” waters, the communication distances are significantly reduced for both photo-sources, with LED-PS reaching a maximum of just 3.39 m for OOK and LD-PS reaching 4.57 m. In this highly turbid environment, both signal attenuation and scattering are much more severe, causing a dramatic reduction in the effective communication range. As the modulation complexity increases, the distances become even shorter, with 64-QAM providing the shortest range at 2.69 m for LED-PS and 3.81 m for LD-PS.

In conclusion, Table 4 illustrates the direct impact of water type, modulation technique, and photo-source on the communication distance in underwater optical wireless communication systems. Simpler modulation schemes Like OOK offer the longest communication distances, especially in clear waters, while more complex schemes such as 64-QAM, while enabling higher data rates, result in reduced ranges. Moreover, the water environment plays a crucial role in determining the range, with turbid environments severely limiting the distance. Therefore, a careful balance between modulation scheme, water type, and photo-source selection is necessary to optimize communication distances in underwater optical communication systems.

**Table 4:** Communication distances for various modulation techniques, water types, and photo-sources at SNR = 30 dB.

Water Type	Modulation Type	LED-PS Distance (m) at SNR = 30 dB	LD-PS Distance (m) at SNR = 30 dB
Pure Seawater	OOK	28.51	55.50
	PPM	26.61	52.78
	DPSK	25.52	51.18
	QPSK	22.88	47.26
	32-PSK	19.24	41.59
	64-QAM	16.66	37.36
Clear Ocean	OOK	20.10	34.81
	PPM	18.99	33.39
	DPSK	18.35	32.56
	QPSK	16.76	30.48
	32-PSK	14.51	27.44
	64-QAM	12.86	25.12
Coastal Ocean	OOK	10.40	15.80
	PPM	9.97	15.30
	DPSK	9.71	15.00
	QPSK	9.07	14.26
	32-PSK	8.15	13.16
	64-QAM	7.44	12.30
Turbid Harbor	OOK	3.39	4.57
	PPM	3.29	4.47
	DPSK	3.23	4.40
	QPSK	3.08	4.24
	32-PSK	2.86	4.00
	64-QAM	2.69	3.81

**Fig. 7:**
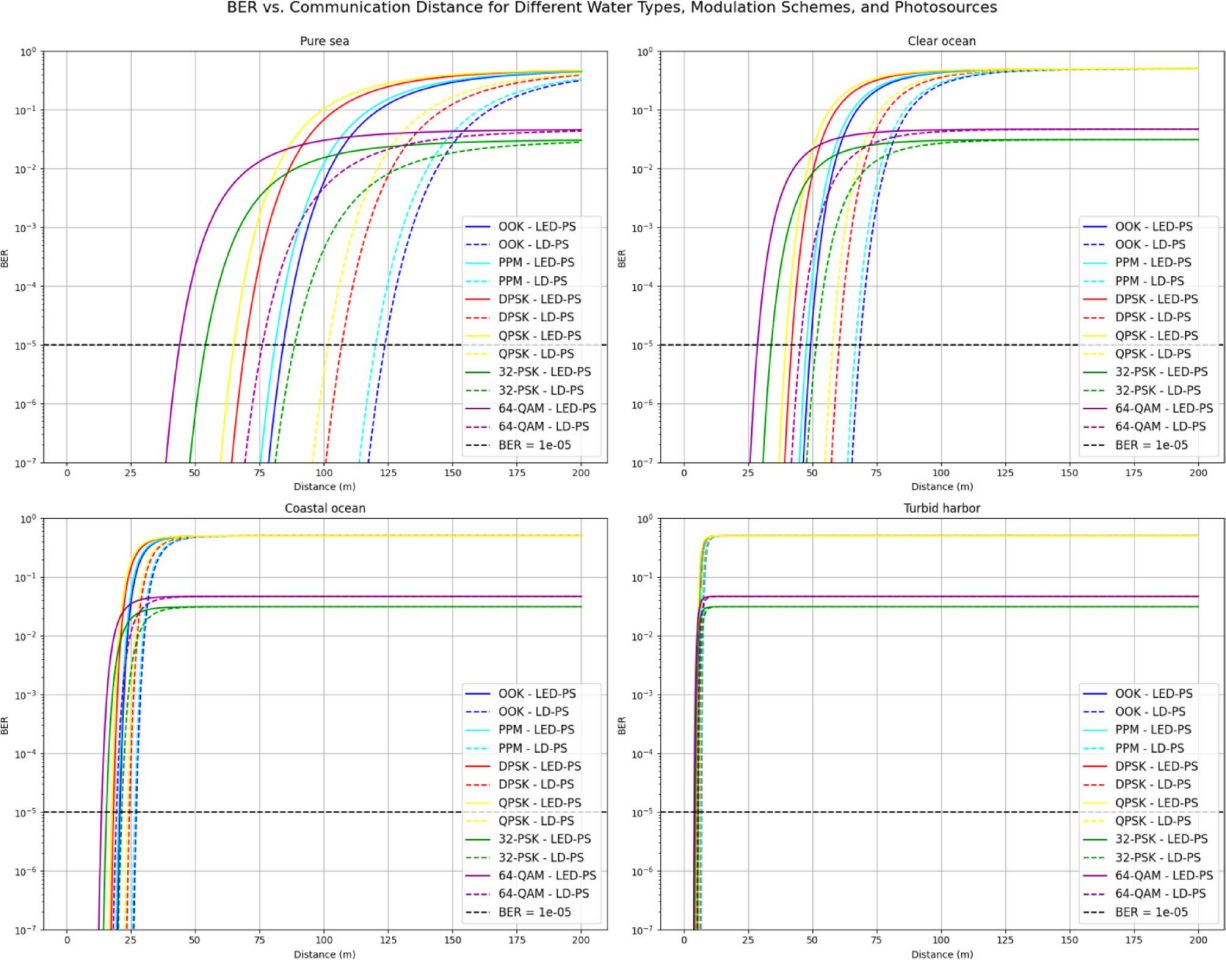
BER vs. communication distance for various water types, modulation techniques, and transmitter types.

Figure 7 illustrates BER as a function of communication distance for the same modulation schemes and water types. The comparison between LED-PS and LD-PS under various attenuation conditions shows increasing BER with distance and performance variation across modulation schemes. A BER threshold of 10⁻⁵ is marked, indicating the maximum effective communication distance for each setup.

Table 5 presents the communication distances achieved by various modulation techniques for different water types and photo-sources (LED-PS and LD-PS) at a BER of 10^−5^. The results clearly highlight the impact of modulation schemes, water clarity, and photo-source type on the achievable communication distances.

- For **pure seawater**, the distances for LED-PS and LD-PS vary significantly across modulation schemes. The OOK modulation provides the longest communication distance for both photo-sources, with values of 84.24 m and 123.73 m for LED-PS and LD-PS, respectively. As the modulation complexity increases, the communication distances decrease, with 64-QAM offering the shortest distances (43.94 m for LED-PS and 76.12 m for LD-PS). This trend is consistent across all water types, where simpler modulation schemes Like OOK and PPM consistently outperform more complex schemes such as 32-PSK and 64-QAM.
- In **clear ocean** conditions, the distances are shorter than those in pure seawater, as expected due to higher water attenuation. For LED-PS, the OOK modulation achieves a distance of 49.33 m, while LD-PS extends the communication distance to 68.52 m. Again, OOK performs the best in terms of distance, with 64-QAM providing the shortest range (28.71 m for LED-PS and 45.30 m for LD-PS). The communication distance for all modulation types in clear ocean conditions is notably reduced compared to pure seawater, reflecting the increased optical attenuation in more turbid water.
- The **coastal ocean** conditions introduce even more attenuation, leading to a further reduction in communication distances. For LED-PS, the OOK modulation achieves a distance of 20.76 m, while for LD-PS, the distance is slightly higher at 27.12 m. As with the previous water types, 64-QAM offers the shortest distances (13.61 m for LED-PS and 19.40 m for LD-PS). These distances indicate the increasing challenge of maintaining communication in more turbid water environments.
- In **turbid harbor** waters, which represent the most challenging conditions for optical wireless communication due to high attenuation, the communication distances are significantly reduced. The OOK modulation achieves a maximum distance of 5.59 m for LED-PS, and 6.85 m for LD-PS. The 64-QAM modulation provides the shortest communication range, with distances of 4.04 m for LED-PS and 5.29 m for LD-PS. These results illustrate the severe impact of water clarity on the performance of underwater optical wireless communication systems, with attenuation becoming a dominant factor at higher turbidity levels.

Overall, the results demonstrate that the communication distance is highly dependent on both the modulation technique and the water type. In clearer waters, simpler modulation schemes Like OOK and PPM provide longer communication distances, while more complex schemes Like 64-QAM and 32-PSK are significantly more affected by water attenuation, leading to shorter achievable distances. Additionally, the type of photo-source (LED-PS vs. LD-PS) also influences the distance, with LD-PS generally offering slightly better performance due to its narrower beam divergence. This comprehensive analysis underscores the importance of selecting the appropriate modulation scheme and photo-source for efficient communication in various underwater environments.

**Table 5:** Communication distances for various modulation techniques, water types, and photo-sources at BER = 10^−5^.

Water Type	Modulation Type	LED-PS Distance (m) at BER = 10^−5^	LD-PS Distance (m) at BER = 10^−5^
Pure Seawater	OOK	84.24	123.73
	PPM	81.02	120.11
	DPSK	69.50	106.93
	QPSK	65.00	101.69
	32-PSK	54.03	88.62
	64-QAM	43.94	76.12
Clear Ocean	OOK	49.33	68.52
	PPM	47.74	66.77
	DPSK	41.97	60.42
	QPSK	39.69	57.88
	32-PSK	34.05	51.49
	64-QAM	28.71	45.30
Coastal Ocean	OOK	20.76	27.12
	PPM	20.22	26.54
	DPSK	18.26	24.46
	QPSK	17.48	23.61
	32-PSK	15.52	21.50
	64-QAM	13.61	19.40
Turbid Harbor	OOK	5.59	6.85
	PPM	5.44	6.78
	DPSK	5.03	6.38
	QPSK	4.86	6.19
	32-PSK	4.46	5.76
	64-QAM	4.04	5.29

**Fig. 8:**
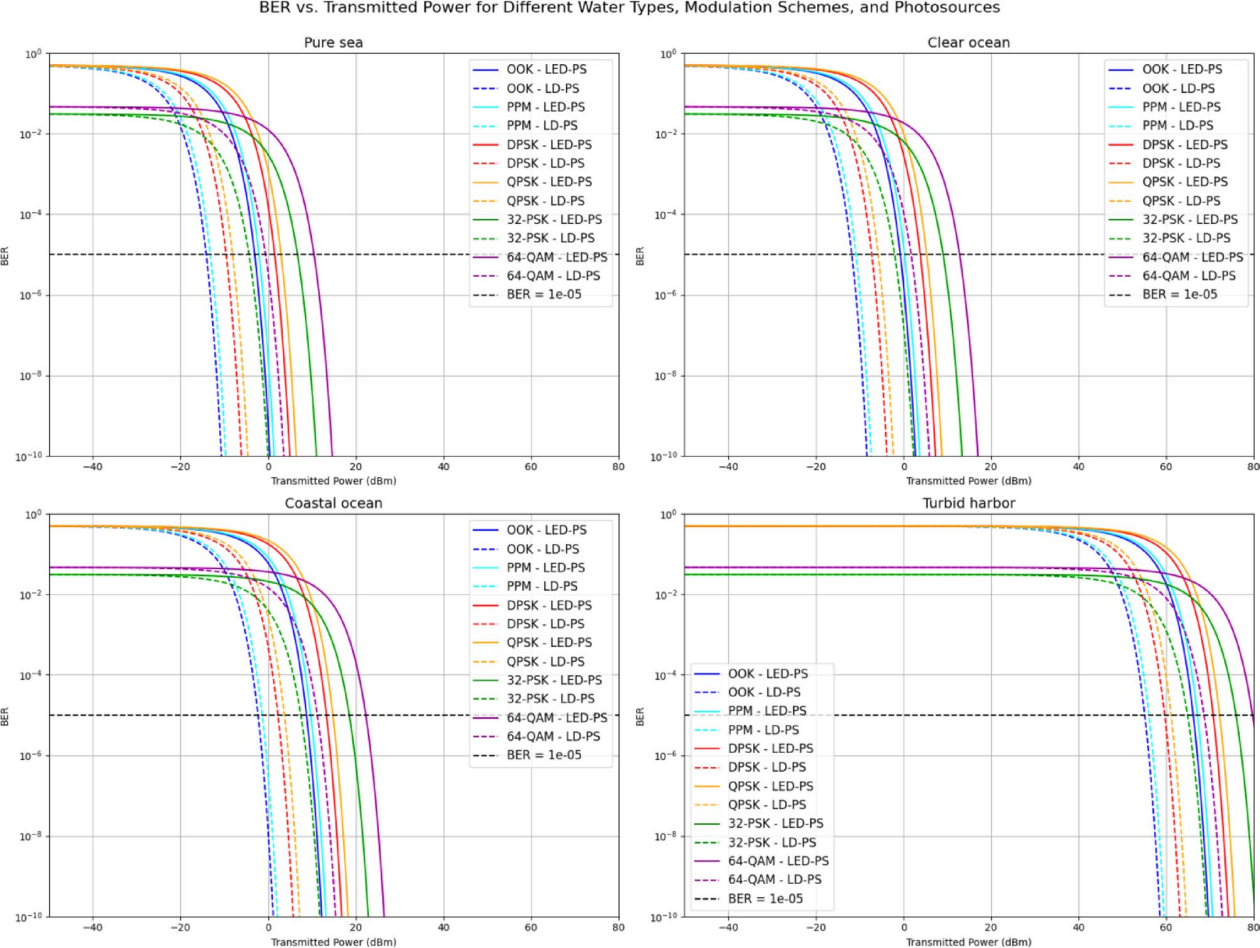
BER vs. transmitted power for various water types, modulation techniques, and transmitter types.

Figure 8 depicts BER versus transmitted power for the modulation schemes using both LED-PS and LD-PS across the underwater environments. Higher-order schemes Like 64-QAM and 32-PSK demand significantly more power to achieve a BER of 10⁻⁵, especially in harsher environments like turbid harbor. In contrast, OOK and PPM achieve the target BER at lower power levels, particularly in pure sea and clear ocean. LD-PS consistently outperforms LED-PS, underlining its efficiency. The results emphasize the trade-off between modulation complexity and power efficiency.

The required transmitted power values for achieving a target BER of 10⁻⁵ across different UOWC scenarios are presented in Table 6. These values offer critical insights into the performance of various modulation techniques when deployed under different water conditions using two types of photo-sources: LED-PS and LD-PS.

- Across all water types, LD-PS consistently outperforms LED-PS by requiring significantly lower transmitted power levels to meet the BER threshold. This advantage is primarily due to the higher directionality and lower beam divergence of laser diodes, which results in reduced optical power losses and enhanced coupling efficiency at the receiver. In **pure seawater**, for instance, the transmitted power required using LD-PS for OOK is −14.11 dBm, whereas LED-PS demands − 3.00 dBm—reflecting a substantial improvement of over 11 dB. A similar trend is evident across other modulation schemes such as 64-QAM, where LD-PS operates at −0.45 dBm compared to 10.51 dBm for LED-PS.
- The performance degradation becomes more pronounced in optically harsher environments such as **turbid harbor water**. In this scenario, both LED-PS and LD-PS require significantly higher transmitted powers to overcome severe absorption and scattering effects. For example, using 64-QAM in turbid waters necessitates a transmitted power of 79.73 dBm for LED-PS and 68.77 dBm for LD-PS—highlighting the impact of channel attenuation on system design. Moreover, the gap between the required power levels for simple modulations (e.g., OOK) and higher-order schemes (e.g., 64-QAM) increases under more turbid conditions, suggesting that higher modulation formats are less suitable in high-attenuation environments without power adaptation mechanisms.
- A comparison across **modulation techniques** reveals that simpler schemes Like OOK and PPM consistently demand lower transmitted powers to achieve the target BER compared to complex modulations such as 32-PSK and 64-QAM. This aligns with theoretical expectations, as higher-order modulations require higher signal-to-noise ratios (SNRs) to maintain reliable performance. In **clear ocean water**, for instance, OOK requires − 0.6 dBm (LED-PS) and − 11.71 dBm (LD-PS), while 64-QAM requires 12.91 dBm and 1.8 dBm, respectively. This difference emphasizes the trade-off between spectral efficiency and energy efficiency in underwater channels.

Overall, the data underscore the importance of selecting appropriate combinations of water type, photo-source, and modulation scheme in UOWC system design. LD-PS paired with lower-order modulations emerges as the most power-efficient configuration, particularly in challenging environments. However, in clearer water conditions where attenuation is minimal, there is more flexibility in adopting higher-order modulation schemes without incurring prohibitive power requirements. These insights are vital for guiding the development of adaptive modulation and power control techniques in future Internet of Underwater Things (IoUT) applications.

**Table 6:** Required transmitted power values (dBm) for various modulation techniques, water types, and photo-sources at BER = 10^−5^.

Water Type	Modulation Type	LED-PS Transmitted power (dBm) at BER = 10^−5^	LD-PS Transmitted power (dBm) at BER = 10^−5^
Pure Seawater	OOK	−3.00	−14.11
	PPM	−1.95	−13.06
	DPSK	1.65	−9.46
	QPSK	3.00	−7.96
	32-PSK	6.76	−4.20
	64-QAM	10.51	−0.45
Clear Ocean	OOK	−0.6	−11.71
	PPM	0.30	−10.81
	DPSK	3.90	−7.21
	QPSK	5.41	−5.71
	32-PSK	9.16	−1.95
	64-QAM	12.91	1.8
Coastal Ocean	OOK	8.86	−2.25
	PPM	9.76	−1.2
	DPSK	13.36	2.40
	QPSK	14.86	3.75
	32-PSK	18.62	7.51
	64-QAM	22.37	11.26
Turbid Harbor	OOK	66.22	55.11
	PPM	67.27	56.16
	DPSK	70.72	59.76
	QPSK	72.22	61.11
	32-PSK	75.98	64.86
	64-QAM	79.73	68.77

**Fig. 9:**
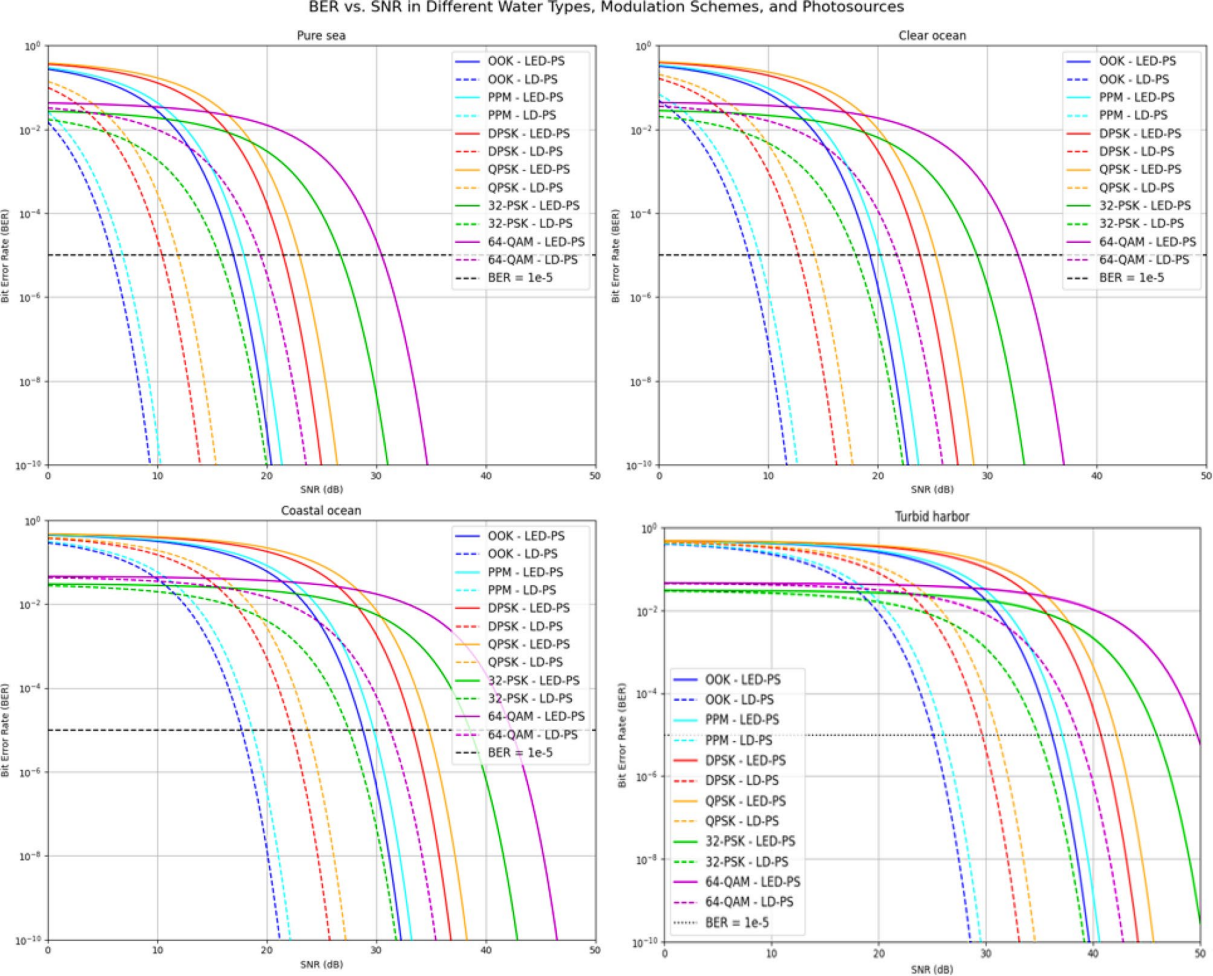
BER vs. SNR for various water types, modulation techniques, and transmitter types.

Figure 9 shows the BER versus SNR for the same modulation schemes and photo sources in all four environments. Higher-order schemes require higher SNR to reach a BER of 10⁻⁵, while simpler schemes like OOK and PPM are more robust. LD-PS again outperforms LED-PS, offering better noise resilience. Performance is best in pure sea and clear ocean, and worst in coastal ocean and turbid harbor. These findings underscore the need to match modulation schemes and sources with environmental conditions to optimize reliability and data rate.

Table 7 presents the required SNR values at a BER of 10⁻⁵ for different modulation techniques, water types, and photo-sources. This table provides insights into the relationship between water clarity, modulation scheme, and photo-source choice in UOWC systems, offering valuable implications for designing efficient communication links in various underwater environments.

- In **pure seawater**, which is characterized by relatively low attenuation and scattering, the SNR values are the lowest across all water types. The required SNR for OOK with LED-PS is 16.95 dB, and with LD-PS, it is significantly lower at 5.88 dB, highlighting the superior performance of LD-PS in this environment. As the modulation scheme increases in complexity, higher SNR values are needed. For instance, 64-QAM requires 30.49 dB with LED-PS and 19.42 dB with LD-PS, demonstrating the greater resilience of simpler modulation techniques, such as OOK and PPM, to noise in pure seawater.
- As we move to **clear ocean** water, the SNR requirements increase due to the higher attenuation and scattering characteristics of this water type. For OOK, the required SNR with LED-PS increases to 19.28 dB, while with LD-PS, it rises to 8.21 dB. Similarly, the required SNR for 64-QAM rises to 32.82 dB with LED-PS and 21.75 dB with LD-PS. This increase in required SNR further underscores the role of water clarity in determining the performance of underwater communication systems. LD-PS continues to outperform LED-PS across all modulation schemes, albeit with a smaller margin than in pure seawater.
- In **coastal ocean** environments, the attenuation becomes more pronounced, leading to even higher SNR values across all modulation schemes. The required SNR for OOK with LED-PS increases to 28.78 dB, and for 64-QAM, it reaches 42.33 dB. The difference between LED-PS and LD-PS becomes more evident, especially at higher-order modulations, with LD-PS consistently requiring lower SNR values. This trend illustrates the growing importance of choosing an efficient photo-source, particularly when dealing with environments that introduce significant optical loss, such as coastal ocean waters.
- The most challenging environment for underwater optical communication, **turbid harbor**, exhibits the highest SNR requirements due to extreme scattering and absorption by suspended particles in the water. For OOK, the required SNR with LED-PS is 36.16 dB, and with LD-PS, it is 25.08 dB. At 64-QAM, the required SNR reaches a staggering 49.70 dB with LED-PS and 38.63 dB with LD-PS. This substantial increase in required SNR emphasizes the need for more robust photo-sources and modulation techniques when operating in turbid waters. The considerable performance advantage of LD-PS over LED-PS is particularly noticeable in these environments, reinforcing the importance of selecting the appropriate photo-source to mitigate the effects of high-water turbidity.

Overall, Table 7 demonstrates a clear trend: as water turbidity increases, the required SNR for reliable communication rises, with higher-order modulations Like 64-QAM and 32-PSK being especially sensitive to changes in water type. The choice of photo-source also plays a critical role, with LD-PS consistently outperforming LED-PS in all water types, particularly in more challenging environments such as turbid harbor waters. These findings highlight the trade-offs between data rate and communication reliability, with higher-order modulations offering higher data rates at the cost of requiring higher SNR, especially in murkier waters. Such insights are crucial for designing adaptive UOWC systems capable of maintaining reliable communication links in varying underwater conditions.

**Table 7:** Required SNR values (dB) for various modulation techniques, water types, and photo-sources at BER = 10^−5^.

Water Type	Modulation Type	LED-PS SNR (dB) at BER = 10^−5^	LD-PS SNR (dB) at BER = 10^−5^
Pure Seawater	OOK	16.95	5.88
	PPM	17.92	6.85
	DPSK	21.51	10.44
	QPSK	22.97	11.90
	32-PSK	26.72	15.65
	64-QAM	30.49	19.42
Clear Ocean	OOK	19.28	8.21
	PPM	20.25	9.18
	DPSK	23.84	12.77
	QPSK	25.30	14.23
	32-PSK	29.05	17.98
	64-QAM	32.82	21.75
Coastal Ocean	OOK	28.78	17.71
	PPM	29.75	18.68
	DPSK	33.34	22.27
	QPSK	34.81	23.73
	32-PSK	38.56	27.49
	64-QAM	42.33	31.26
Turbid Harbor	OOK	36.16	25.08
	PPM	37.13	26.05
	DPSK	40.72	29.64
	QPSK	42.18	31.11
	32-PSK	45.93	34.86
	64-QAM	49.70	38.63

**Fig. 10:**
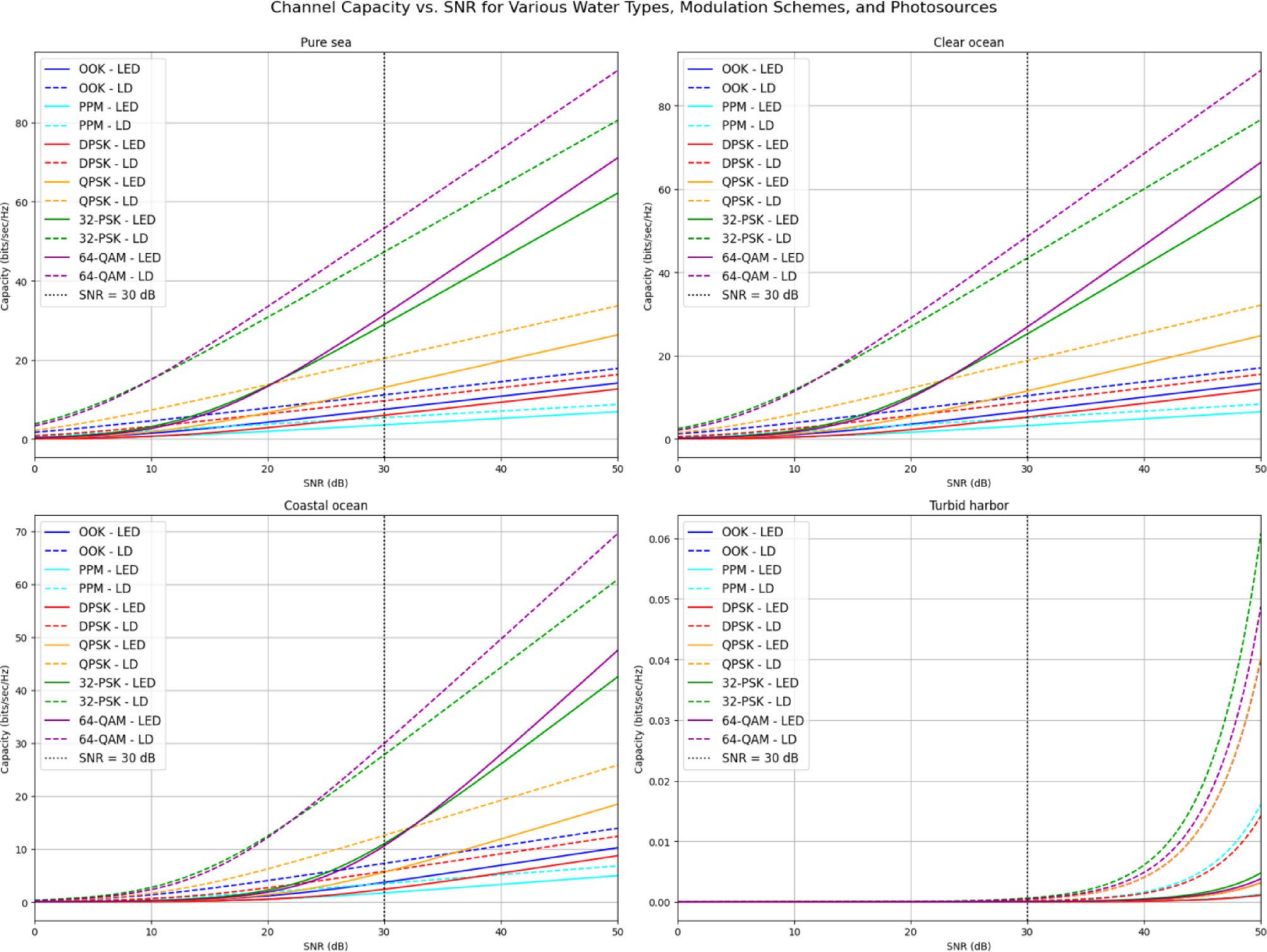
Shannon spectral efficiency (upper bound) vs. SNR for various water types, modulation techniques, and transmitter types.

Figure 10 displays Shannon spectral efficiency (upper bound) versus SNR for different underwater environments. In pure sea and clear ocean, high-order modulation schemes (64-QAM, 32-PSK) deliver higher capacity at elevated SNR levels. In contrast, coastal ocean and especially turbid harbor show degraded capacity due to increased scattering and absorption, with the latter falling below 0.06 bits/sec/Hz. Across all conditions, LD-PS outperforms LED-PS, affirming its superior efficiency. These results highlight the balance between modulation complexity and environmental limitations in underwater optical communication.

Table 8 presents the Shannon spectral efficiency (upper bound) values (in bits per second per Hertz) for various modulation techniques, photo-sources, and water types at an SNR of 30 dB. These results provide critical insights into the performance of optical wireless communication systems in different underwater environments, taking into account the impact of modulation schemes and the type of photo-source used.

- At **Pure Seawater**, the channel capacities for all modulation techniques are relatively high, especially for the LED-PS and LD-PS configurations. For example, the 64-QAM modulation achieves a significant channel capacity of 31.38 bps/Hz for LED-PS and 53.23 bps/Hz for LD-PS, reflecting the high efficiency of this scheme in clear water conditions. Similarly, other higher-order modulation schemes Like 32-PSK also show strong performance, with capacities of 29.01 bps/Hz for LED-PS and 47.28 bps/Hz for LD-PS. In contrast, lower-order modulations such as OOK and PPM exhibit much lower capacities, with OOK achieving 7.52 bps/Hz for LED-PS and 11.19 bps/Hz for LD-PS. The presence of these variations highlights how higher-order modulations benefit from the clearer signal propagation in pure seawater.
- Moving to the **Clear Ocean**, the overall channel capacity values are slightly reduced compared to pure seawater. However, the trend remains consistent across modulation techniques, with 64-QAM and 32-PSK continuing to provide the highest capacities. For instance, 64-QAM achieves 26.90 bps/Hz for LED-PS and 48.61 bps/Hz for LD-PS. As in pure seawater, the lower-order modulation techniques such as OOK and PPM still provide significantly lower channel capacities, further emphasizing the advantage of using higher-order modulations for maximizing throughput.
- In the **Coastal Ocean**, the channel capacities decrease further due to the increased scattering and absorption effects inherent in coastal water conditions. Here, 64-QAM provides a channel capacity of 10.58 bps/Hz for LED-PS and 29.91 bps/Hz for LD-PS, which is considerably lower than the capacities observed in pure seawater and clear ocean conditions. This reduction is observed across all modulation techniques, with OOK and PPM showing minimal values, indicating that these modulation techniques are poorly suited for such challenging environments.
- Finally, in the **Turbid Harbor**, the channel capacities drop to near zero for all modulation techniques. The extremely high attenuation and scattering in turbid waters significantly Limit the communication range and capacity, rendering most modulation schemes ineffective. OOK and PPM have channel capacities of 0 and 0.0004 bps/Hz for LED-PS, respectively, while 64-QAM has a mere 0.0005 bps/Hz for LD-PS. These near-zero capacities underscore the harsh impact of turbidity on optical wireless communication, where the scattering and absorption in such environments make high-capacity transmission infeasible.

In summary, the data in Table 8 demonstrate the strong correlation between water type and channel capacity. Clearer water types Like Pure Seawater and Clear Ocean support higher channel capacities, particularly for high-order modulations such as 64-QAM and 32-PSK. In contrast, Coastal Ocean and Turbid Harbor conditions result in progressively lower channel capacities, making optical communication increasingly unreliable. This underscores the importance of choosing appropriate modulation techniques and understanding the environmental factors at play when designing underwater optical wireless communication systems.

**Table 8:** Shannon spectral efficiency (upper bound) values (bps/Hz) for various modulation techniques, water types, and photo-sources at SNR = 30 dB.

Water Type	Modulation Type	LED-PS channel capacity (bps/Hz) at SNR = 30 dB	LD-PS channel capacity (bps/Hz) at SNR = 30 dB
Pure Seawater	OOK	7.5211	11.1919
	PPM	3.6006	5.4351
	DPSK	6.0211	9.6785
	QPSK	13.0580	20.3851
	32-PSK	29.0120	47.2819
	64-QAM	31.3818	53.2347
Clear Ocean	OOK	6.7536	10.4192
	PPM	3.2175	5.0488
	DPSK	5.2636	8.9066
	QPSK	11.5337	18.8405
	32-PSK	25.2373	43.4235
	64-QAM	26.9049	48.6089
Coastal Ocean	OOK	3.6980	7.2698
	PPM	1.7018	3.4751
	DPSK	2.3764	5.7724
	QPSK	5.6102	12.5581
	32-PSK	10.9980	27.7722
	64-QAM	10.5822	29.9085
Turbid Harbor	OOK	0	0.0004
	PPM	0	0.0002
	DPSK	0	0.0001
	QPSK	0	0.0004
	32-PSK	0	0.0006
	64-QAM	0	0.0005

Table 9 illustrates the trade-offs between the six selected modulation schemes, visually emphasizing their respective roles and differences. The spectral efficiency values in Table 9 were computed based solely on modulation symbol rate assumptions (e.g., $\:{log}_{2}\left(M\right)$).

**Table 9:** Comparative trade-off analysis of the proposed modulation schemes inUOWC.

Modulation	Type	Spectral Efficiency	Power Efficiency	Complexity	Relevance in UOWC
OOK	Intensity	Low (1 bps/Hz)	High	Very Low	Common baseline; widely used
PPM	Intensity (Pulse-based)	Low (≤ 1 bps/Hz)	Very High	Low	Preferred in low-power UOWC
DPSK	Phase	Moderate (2 bps/Hz)	Moderate	Moderate	Robust against phase distortion
QPSK	Phase	Moderate (2 bps/Hz)	Moderate	Moderate	Balance between capacity and complexity
32-PSK	Phase	High (> 5 bps/Hz)	Lower	High	Higher spectral efficiency
64-QAM	Amplitude & Phase	Very High (~ 6 bps/Hz)	Lower	Very High	Benchmarks capacity in clear conditions

Based on the quantitative analysis, the selection of modulation schemes in UOWC systems should be aligned with the specific environmental conditions and performance requirements of the intended application. Table 10 provides a summarized set of design guidelines to support optimal decision-making.

**Table 10:** Design guidelines Summary.

Application Type	Recommended Modulation	Conditions	Key Metrics (LD-PS, BER = 10⁻⁵)
Long-range sensing	OOK	Pure/clear waters	Distance: 123.73 m; SNR: 5.88 dB; Power: − 14.11 dBm
Energy-constrained nodes	PPM	Moderate environments	Distance: ~66 m; SNR: ~6.85 dB; Spectral Eff.: 5.43
High-throughput short-range	64-QAM	Clear/pure waters	Capacity: 53.23 bps/Hz; Range: ~76 m; SNR: 19.4 dB
Harsh/turbid conditions	OOK or fallback to RF	Turbid harbor	Distance: <7 m; Power: >55 dBm; Capacity: <0.001 bps/Hz

**Finally**, Table 11 provides a concise comparison of the previous studies alongside our current work, focusing on the modulation techniques, key findings, and performance metrics across various water types and conditions. It also highlights the main advantages and limitations of each approach, offering a clear perspective on how different modulation strategies perform under diverse underwater communication scenarios.

**Table 11:** A concise comparison of literature reviews alongside our current work on UOWC systems.

Study	Modulation Techniques	Key Findings	Performance Metrics	Water Types & Conditions	Key Advantages/Limitations
^19^	PPM, PSK, OOK, DPSK, FSK	PPM is suited for low power, PSK offers bandwidth but poor power efficiency, OOK has power and error limitations, DPSK offers strong error resilience but high power, FSK is power-intensive	Bandwidth, Error Performance, Power Efficiency	Various Water Types	Emphasizes the need to balance modulation technique and water conditions for optimal performance
^20^	PPM, DPIM, OOK	PPM is energy-efficient, DPIM has better bandwidth efficiency but higher complexity, OOK is less suitable for energy-constrained environments	Energy Efficiency, Bandwidth Efficiency, Computational Complexity	Realistic System Parameters	Highlights trade-offs between energy efficiency, bandwidth, and computational complexity
^21^	H-QAM, QAM	H-QAM performs well in clear waters, Si APD outperforms Ge APD in low-chlorophyll environments, chlorophyll reduces power and SNR	Signal Reception, Transmission Distance, SNR, BER	Jerlov Water Types	QAM outperforms others, and Si APD provides better performance in low-chlorophyll water
^22^	OOK, 2DPSK	OOK and 2DPSK perform better in Jerlov Type I water at 450 nm, increased chlorophyll reduces performance	Transmission Distance, SNR, Power	Jerlov Type I, Chlorophyll Variations	450 nm wavelength is optimal for underwater communication
^23^	OOK, QPSK, OFDM	OFDM offers superior performance with lower BER and higher bandwidth efficiency under turbulence	BER, Bandwidth Efficiency	Various Water Types, Turbulence	OFDM is preferable for low-energy transmissions with acceptable BER
^24^	PSK, DPSK, PAM, QAM	QAM offers the best performance with lower BER across all water conditions	BER	Pure Seawater, Turbid Harbor Water	QAM is best for underwater free-space optical communication
^25^	M-PSK, M-QAM	Heterodyne detection outperforms IM/DD, M-PSK achieves better BER than M-QAM	BER, System Performance	Various Water Types, Turbulence	Receiver aperture size and beam alignment significantly improve performance
^26^	4-QAM	4-QAM achieves the best BER performance in pure seawater with high power requirements in clear ocean waters	BER, Power Requirements	Pure Seawater, Clear Ocean	4-QAM provides excellent performance, but power increases in clearer waters
^27^	SIM, PPM	SIM and PPM are resilient to turbulence, SIM achieves higher data rates	Data Rates, BER	Still and Turbulent Water	SIM is superior in still water, PPM resilient in turbulence
^28^	DQPSK, OFDM-QAM	DQPSK outperforms other schemes in eye-opening and link reach, OFDM-QAM is effective at long distances	Eye-Opening, Q-factor, Link Reach	Deep Water	DQPSK is robust against phase shifts and fading; OFDM-QAM is ideal for long distances
^29^	64-QAM, 32-PSK	CNN-based modulation recognition achieves 100% accuracy, outperforming traditional methods	Accuracy, Precision, Recall, F1-Score	Various Water Types, Turbulence	CNN enhances IoUT communication reliability and performance
^30^	64-QAM, 32-PSK, OOK	MIMO outperforms SISO and SIMO, showing significant improvements in data rate, communication range, and reliability	Data Rate, Power Reduction, Channel Capacity	Pure and clear water, Turbulence	MIMO offers superior performance in challenging underwater environments, ideal for IoUT
^31^	Dual-polarization 16-QAM + OFDM	Achieved 80 Gbps with orthogonal polarization; range up to 13.93 m in low-attenuation water, ~ 3 m in Harbor I.	BER, EVM, Constellation Diagrams	10 water types including pure, Jerlov I, Harbor I	High capacity; limited range in turbid waters due to attenuation.
^32^	NRZ, AMI, CSRZ	NRZ outperformed AMI and CSRZ; max 28 m in pure sea (NRZ), min 3.96 m in harbor II (CSRZ).	BER, Q-Factor, Received Power, Eye Diagrams	PS, CO, CS, HI, HII	NRZ had best BER and range; CSRZ worst performance in all waters.
^33^	NRZ-OOK, PPM, QAM-CAP, OFDM	OFDM and space-domain index modulation showed best spectral efficiency and BER; supported > 30 Gbps over 21 m.	BER, Spectral Efficiency	Turbulent conditions, LoS/NLoS setups	Advanced modulations improve robustness; challenges with channel modeling and energy efficiency remain.
^34^	OAM Multiplexing (LG₀,₀, LG₀, ₂₀, LG₀,₅₀ at 532 nm)	Achieved 30 Gbps using 3 OAM beams; longest range of 22 m (JI), shortest 4.2 m (JIII); all links met Q > 4, log (BER) < − 5.	BER, Q-Factor, Eye Diagrams	Jerlov I–III	High capacity via OAM; performance depends heavily on water attenuation.
^35^	OCDMA with Fixed Right Shift (FRS) Code	Achieved 30 Gbps using 3 channels; range up to 35 m (JI) at 10 Gbps/channel; performance degrades with higher data rates and more attenuating waters.	BER, Q-Factor, Eye Diagrams	Jerlov I–III, varying chlorophyll concentrations	Supports parallel transmission; affected by water type and rate scaling.
^36^	Space Division Multiplexing (HG Modes) + NRZ (20 Gbps x 4)	Realized 80 Gbps over 1800–1950 m; 850 nm wavelength and 50 μm aperture optimized performance under high attenuation.	BER, Link Range	High attenuation waters, varied wavelengths	Very long-range UOWC; spatial multiplexing boosts capacity; sensitive to wavelength and aperture optimization.
**The current work**	**OOK**,** PPM**,** DPSK**,** QPSK**,** 32-PSK**,** and 64-QAM**	**OOK and PPM offer longer ranges with lower power and SNR needs; 32-PSK and 64-QAM provide higher capacity but require higher power and SNR. LD-PS outperforms LED-PS. Performance declines significantly in turbid waters.**	**Received Power**,** BER**,** SNR**,** Channel Capacity**,** Communication Range**	**Pure**,** Clear**,** Coastal**,** and Turbid Waters under different turbulence and attenuation levels**	**Comprehensive evaluation across modulation types**,** water conditions**,** and source types (LED/LD**,** SiPM-PD). Highlights trade-offs between range and efficiency. Limitation: degraded performance in turbid waters signals need for adaptive modulation.**

### Benchmarking and experimental validation

While our study is based on simulations, several experimental works have established valuable benchmarks that align with and extend our findings. For example, researchers have characterized LD-based UOWC links using water tank setups, quantifying optical losses over distances up to several meters^45^. MLSE-enhanced OOK experiments combined with simulations have validated bandwidth limitation models in laboratory environments ^46^. Polarization multiplexing techniques demonstrated data transmission up to 92 m using photon-counting detection^47^, while modulation schemes like SIM showed enhanced resilience under turbulent conditions ^48^. Additionally, high data rate transmissions (up to 25 Gbps) over short distances and AOM-based communication over 18 m have been experimentally validated^49,50^. Turbulence-induced fading modeled via log-normal distributions was confirmed in lab-scale water pool measurements ^51^; BER and sensitivity were assessed under varied turbidities using LED and PMT setups^52^; and alignment sensitivity and geometric losses were quantified in LED vs. laser propagation studies in controlled tanks ^53^. FPGA-implemented LD systems operating at 50 Mbps under moderate conditions achieved BER below FEC limits^54^. Notably, Wang et al. achieved 100 m/500 Mbps UOWC in laboratory settings with acceptable BER^50^. Meanwhile, He et al. investigated salinity-driven turbulence effects on LD-based UOWC ^55^. These experimental studies underscore the practical feasibility of UOWC systems across varied conditions and directly inform the planned extensions of our work. In future research, we will use these empirical benchmarks to validate and refine our simulation framework, conduct laboratory tank experiments, and pursue field deployments to strengthen the generalizability and reliability of our results. Table 12 compares recent experimental validation studies with the present simulation-based framework. While prior works have provided valuable empirical demonstrations of specific modulation schemes or system components, our study differs by offering a unified, multi-parameter performance comparison of six widely adopted modulation schemes under diverse environmental and system conditions. This makes the present work complementary to experimental efforts, as it provides a baseline framework that can guide both future experiments and system design for IoUT applications.

**Table 12:** Comparison between existing practical validation studies and the present Work.

Ref./Study	Setup & Modulation	Distance & Environment	Key Findings	Relation to Present Work
^45^ Blue LD under practical conditions	450 nm blue LD, NRZ-OOK	6 m in tap water; 3 m in artificial seawater	BER < 10⁻⁸ at 1.25 Gbps in tap water; performance degraded in seawater due to scattering; temperature significantly affected BER	Provides real-world insight into environmental impacts (temperature, salinity, turbulence). Our work generalizes by modeling multiple water types and turbulence statistically.
^46^ Bandwidth limitations & MLSE	OOK with MLSE	Up to 12 m, harbor water	MLSE extended bitrate from 2.4 Gbps to 4 Gbps (experiment); improved simulated BER and reduced power budget in harbor water	Demonstrates equalization benefits in bandwidth-limited setups. Our study complements by comparing six modulation schemes under varied noise and turbulence conditions.
^47^ Polarization multiplexing with photon counting	Polarization multiplexing OOK & 2-PPM with photon-counting	92 m, seawater channels (Jerlov I, IA, IB)	Achieved 14.58 Mbps (pol. OOK) and 7.29 Mbps (pol. 2-PPM); potential reach 264 m at c = 0.07 m⁻¹; BER reduction with polarization multiplexing	Highlights advanced modulation and polarization gains. Our work complements by benchmarking classical modulations across conditions, providing a baseline for such advanced techniques.
^48^ OOK vs. SIM under turbulence	OOK & SIM (including PSK-SIM, QAM-SIM)	Gbps links; tested in still and turbulent water	SIM offered resilience to turbulence; achieved up to 4.2 Gbps (16-QAM-SIM)	Validates turbulence mitigation with SIM. Our study captures turbulence impacts analytically and compares trade-offs across schemes.
^49^ 25 Gbps VCSEL in turbid harbor water	680 nm VCSEL + beam expander, NRZ-OOK	5 m, turbid harbor water	25 Gbps with BER = 3 × 10⁻⁹; error-free real-time transmission	Demonstrates high-speed links in harsh water. Our study offers a broader, multi-environment framework beyond single setup optimization.
^50^ AOM-based green LD system	520 nm LD + AOM, NRZ-OOK	9–18 m, tap water and turbid suspensions	BER 1.53 × 10⁻⁴ (9 m, 15 Mbps); BER 4.7 × 10⁻³ (18 m, 12 Mbps)	Shows mid-range feasibility of AOM-based UWOC. Our study explores trade-offs across wider scenarios and water conditions.
^51^ Turbulence statistics	Experimental turbulence setup; BER and scintillation measured	Variable link spans; turbulence induced by temp/flow	Measured PDFs followed lognormal fading; confirmed turbulence-induced power loss	Validates turbulence models used in simulations. Our study applies these models systematically across six modulations.
^52^ High sensitivity LED + PMT	Blue LED + PMT receiver, RZ-OOK	Up to 100 m in clear seawater	Sensitivity down to − 76 dBm; achieved 1–5 Mbps up to 100 m	Demonstrates LED-based long-reach feasibility. Our study compares LED vs. LD comprehensively under varying conditions.
^53^ Light source & water type	LEDs (RGB) vs. LD, multiple water types	Glass chamber; clear, sea, and cloudy water	Showed differences in geometric loss & attenuation; LD outperformed LEDs	Confirms LED vs. LD trade-offs. Our study integrates these effects systematically in simulations.
^54^ FPGA-based 16-QAM	450 nm LD, 16-QAM on FPGA	3 m in artificial seawater	50 Mbps with BER = 7.11 × 10⁻⁴ (below FEC limit)	Demonstrates moderate-rate LD feasibility. Our study provides a broader comparative framework across six modulations.
^55^ Salinity-induced turbulence	520 nm LD under saline turbulence	Pure vs. saline water	Salinity-induced turbulence reduced optical power; BER nearly unaffected	Provides practical turbulence validation. Our study analytically models turbulence effects in multi-scheme comparison.
**This Work (Comprehensive simulation-based evaluation of modulation schemes)**	**OOK**,** PPM**,** QPSK**,** DPSK**,** 32-PSK**,** 64-QAM**	**Pure**,** clear**,** coastal**,** and turbid water; turbulence modeled; LED-PS and LD-PS**	**Comprehensive performance comparison (BER**,** SNR**,** capacity**,** range) across multiple environments**	**Extends beyond isolated experiments by offering a unified framework and systematic benchmarking for UOWC system design.**

As shown in Table 12, experimental works have achieved impressive performance milestones—ranging from multi-Gbps LD-based transmissions in clear water to long-range LED-based links with photon-counting detectors, as well as turbulence-resilient approaches using SIM or polarization multiplexing. These studies validate the feasibility of UWOC under specific conditions but are often constrained to narrow setups and modulation choices. In contrast, the present study systematically integrates multiple environmental conditions, turbulence models, and two distinct photo-sources (LED and LD) into a unified comparative framework across six modulation schemes. This broader perspective provides generalized insights and performance baselines, complementing experimental findings and supporting practical design and optimization of future IoUT systems.

### Implementation challenges in LD-based UOWC systems

While LD-PS transmitters provide distinct advantages in terms of high directionality, improved optical efficiency, and extended communication reach compared with LED-based sources, they also present critical implementation challenges that must be carefully addressed in practical UOWC deployments. Two of the most significant challenges are transmitter–receiver alignment sensitivity and turbulence-induced distortions.

- Alignment Sensitivity

Unlike LED-based systems that benefit from wider beam coverage and relaxed pointing requirements, LD-based systems are highly sensitive to transmitter–receiver misalignment ^45, 53^. Even small angular or lateral deviations can cause substantial reductions in received optical power and link stability. This sensitivity arises from the narrow beam divergence of LDs, which, while enabling longer reach and higher received power under ideal conditions, also makes them vulnerable to dynamic misalignment induced by water currents, platform mobility, or mechanical vibrations ^51, 55^. Experimental studies, including both water tank and field trials, have confirmed this challenge, demonstrating that LD links often require mitigation strategies such as beam expanders^49^, multi-aperture receiver designs, or adaptive pointing and tracking mechanisms. However, while these techniques can enhance robustness, they also add complexity and cost, underscoring the trade-off between alignment requirements and the practical constraints of underwater deployments.

(b) Turbulence-Induced Distortions:

Another major challenge in LD-based UOWC systems arises from turbulence-induced fading, including beam wandering, scintillation, and power fluctuations ^45, 51, 55^. Unlike LED-PS links, which benefit from wider beam coverage and spatial averaging, narrow-beam LD-PS links are far more vulnerable to turbulence effects ^45, 48^. These impairments stem from random refractive index fluctuations caused by temperature gradients, salinity variations, and flow dynamics, leading to significant degradation in received signal strength and increased bit error rate (BER). Experimental investigations, both in controlled water tanks and natural environments, have confirmed that turbulence can severely impact LD-based link stability, particularly in turbid or high-salinity waters ^45, 48, 55^. While turbulence is often modeled statistically using log-normal or gamma-gamma fading distributions ^51^, its dynamic and spatially variable nature in practice makes reliable compensation challenging. To mitigate these effects, several approaches have been explored, including forward error correction (FEC), adaptive optics, and hybrid optical–acoustic designs; however, such countermeasures often introduce additional complexity and cost, leaving turbulence a persistent bottleneck for stable LD-based underwater communication.

The findings of this study provide a simulation-based foundation for evaluating the performance of LD-based systems under turbulence and misalignment conditions. However, alignment sensitivity and turbulence-induced distortions remain practical bottlenecks that require further investigation beyond mathematical modeling. Future work will therefore focus on experimental validation through controlled water-tank setups and field trials, allowing direct quantification of misalignment penalties and turbulence dynamics. In addition, the integration of adaptive modulation, AI-driven beam tracking and optimization, hybrid communication strategies, and advanced signal processing techniques will be explored to enhance robustness and mitigate these challenges in real-world LD-based UOWC deployments.

## Conclusions

This study conducted a comprehensive quantitative evaluation of six key modulation techniques—OOK, PPM, DPSK, QPSK, 32-PSK, and 64-QAM—within the framework of UOWC systems. Simulations were performed across a range of environmental conditions (pure seawater, clear ocean, coastal ocean, and turbid harbor) and hardware configurations (LED-based and LD-based photo-sources), focusing on metrics such as communication distance, required transmit power, BER, SNR, and channel capacity. Our results confirm established trade-offs but also provide quantitative performance boundaries and application-specific guidelines. For example:

- Long-range, energy-limited applications (e.g., environmental monitoring) benefit most from OOK with LD-PS, achieving up to 123.73 m at BER = 10⁻⁵ in pure seawater, with a required SNR as low as 5.88 dB, and transmit power below - 14.11 dBm.
- For mid-range communications in moderate environments (e.g., clear ocean), PPM offers a Balanced trade-off, providing over 66 m at BER = 10⁻⁵ with LD-PS and moderate power and SNR demands.
- In contrast, high-data-rate, short-range systems (e.g., underwater robotics or real-time video streaming) should consider 64-QAM, which reached a peak Shannon spectral efficiency of 53.23 bps/Hz at SNR ≥ 30 dB, albeit with a reduced communication distance (~ 76 m) and higher transmit power requirements.
- The performance gap between LD-PS and LED-PS was also quantified, showing 11–13 dB power savings and 2× range improvement for LD-PS, making it a superior choice for bandwidth-sensitive or power-limited deployments.

Additionally, a performance degradation model was observed with increasing water turbidity: in turbid harbor water, even OOK and PPM dropped below 7 m in range and required power levels exceeding 55 dBm. Under such conditions, adaptive modulation schemes or hybrid acoustic-optical systems may be necessary to maintain communication. In conclusion, this work moves beyond general trade-offs by establishing quantitative, environment-specific benchmarks and providing targeted guidance for modulation scheme selection in UOWC systems.

## Future work directions

While this study provides a comprehensive evaluation of modulation schemes in UOWC systems, several areas warrant further investigation to enhance system performance and adaptability. Future research should explore:

1. **Adaptive Modulation Techniques**: Implementing real-time adaptive modulation strategies that dynamically switch between different schemes based on environmental conditions and channel state information to maximize efficiency.
2. **Machine Learning-Based Optimization**: Leveraging deep learning models to predict channel characteristics and optimize modulation selection for improved BER and SNR performance in varying underwater conditions.
3. **Experimental Validation**: Conducting real-world experiments in controlled underwater environments to validate the theoretical and simulated results, ensuring practical applicability in operational UOWC systems [44].
4. **Multi-Hop and Cooperative Communication**: Investigating the impact of multi-hop communication and relay-assisted transmission on modulation performance to extend communication range and mitigate signal attenuation in deep-sea environments.
5. **Hybrid Modulation and Coding Schemes**: Exploring hybrid modulation techniques that combine multiple schemes (e.g., QPSK with adaptive power allocation) to enhance both spectral and power efficiency while maintaining robustness against turbulence and scattering.
6. **Integration with IoUT and Underwater Networks**: Examining how modulation techniques can be optimized for large-scale Internet of Underwater Things (IoUT) applications, integrating UOWC with acoustic and RF communication networks for seamless underwater data transmission.
7. **Impact of Water Chemistry and Biological Factors**: Analyzing the effects of varying salinity, temperature gradients, and biological particles on modulation performance to improve UOWC system adaptability in diverse oceanic environments.
8. **A dedicated sensitivity analysis of optical and receiver hardware parameters**: including the variation in the transmitter divergence angle, semi-angle at half power, photodetector responsivity, and aperture size.

## Data Availability

The datasets used and/or analysed during the current study available from the corresponding author on reasonable request.
